# The novel ECF56 SigG1-RsfG system modulates morphological differentiation and metal-ion homeostasis in *Streptomyces tsukubaensis*

**DOI:** 10.1038/s41598-020-78520-x

**Published:** 2020-12-10

**Authors:** Rute Oliveira, Matthew J. Bush, Sílvia Pires, Govind Chandra, Delia Casas-Pastor, Georg Fritz, Marta V. Mendes

**Affiliations:** 1 Bioengineering and Synthetic Microbiology Group, i3S- Instituto de Investigação e Inovação em Saúde, Universidade do Porto, Porto, Portugal; 2 IBMC, Instituto de Biologia Molecular e Celular, Universidade do Porto, Porto, Portugal; 3 Programa Doutoral em Biologia Molecular e Celular (MCBiology), ICBAS, Instituto de Ciências Biomédicas Abel Salazar, Universidade do Porto, Porto, Portugal; 4 Department of Molecular Microbiology, John Innes Centre, Norwich Research Park, Norwich, NR4 7UH UK; 5 Center for Synthetic Microbiology, Philipps-University Marburg, 35032 Marburg, Germany; 6 Present Address: Jill Roberts Institute for IBD Research, Weill Cornell Medicine, New York, NY 10021 USA; 7 School for Molecular Sciences, University of Western Australia, Perth, 6009 Australia

**Keywords:** Microbiology, Bacterial physiology, Bacterial transcription

## Abstract

Extracytoplasmic function (ECF) sigma factors are key transcriptional regulators that prokaryotes have evolved to respond to environmental challenges. *Streptomyces tsukubaensis* harbours 42 ECFs to reprogram stress-responsive gene expression. Among them, SigG1 features a minimal conserved ECF σ_2_–σ_4_ architecture and an additional C-terminal extension that encodes a SnoaL_2 domain, which is characteristic for ECF σ factors of group ECF56. Although proteins with such domain organisation are widely found among Actinobacteria, the functional role of ECFs with a fused SnoaL_2 domain remains unknown. Our results show that in addition to predicted self-regulatory intramolecular amino acid interactions between the SnoaL_2 domain and the ECF core, SigG1 activity is controlled by the cognate anti-sigma protein RsfG, encoded by a co-transcribed *sigG1*-neighbouring gene. Characterisation of ∆*sigG1* and ∆*rsfG* strains combined with RNA-seq and ChIP-seq experiments, suggests the involvement of SigG1 in the morphological differentiation programme of *S. tsukubaensis*. SigG1 regulates the expression of alanine dehydrogenase, *ald* and the WhiB-like regulator, *wblC* required for differentiation, in addition to iron and copper trafficking systems. Overall, our work establishes a model in which the activity of a σ factor of group ECF56, regulates morphogenesis and metal-ions homeostasis during development to ensure the timely progression of multicellular differentiation.

## Introduction

Streptomycetes are unique soil-dwelling bacteria with a prolific biosynthetic capacity responsible for hundreds of widely used bioactive compounds. The onset of streptomycetes specialized metabolism and the production of secondary metabolites is intimately related with the morphological differentiation process.

*Streptomyces* species follow a complex multicellular filamentous lifecycle that starts with the germination of a spore when the environmental conditions are favourable. Hyphae emerge that grow by tip-extension and branching, to form a dense filamentous vegetative mycelium that facilitates nutrient scavenging. When nutrients are depleted, a highly regulated process of differentiation is initiated. Vegetative growth is halted before the erection of aerial hyphae that extend upwards, away from the vegetative mycelium. Previously, we demonstrated that differentiation in *S. natalensis* is tightly controlled by oxidative stress^1^. We illustrated that the transition from first mycelium stage (MI) to secondary metabolite producing differentiated mycelium (MII) is preceded by phenomena of cell death in the vegetative mycelia, which reroutes nutrients to differentiate MII hyphae into nascent aerial mycelium. Also, pivotal to this substrate to aerial mycelium transition is the production of a hydrophobic coat that consists of two families of proteins, the rodlins and the chaplins^2,3^. Subsequently, cell division and chromosome segregation generate chains of uni-genomic spores. Fully mature spores frequently acquire a strain-specific colour derived from a polyketide spore pigment produced during the late stages of development. The process of differentiation in the *Streptomyces* genus has been largely studied in strains used as model organisms such as *S. coelicolor* and *S. venezuelae.* It is strictly dependent on gene networks which are controlled by the Bld (bald) and the Whi (white) family of regulators^4–6^. The Bld proteins orchestrate the transition to the aerial lifestyle and so disruption of *bld* genes blocks the erection of aerial mycelium (a “bald” phenotype). The Whi proteins mediate the conversion of aerial hyphae into spores and so disruption of *whi* genes results in a block at the aerial growth stage (a “white” phenotype).

In order to survive complex niches, streptomycetes have developed sophisticated response mechanisms to environmental stress signals. Many such mechanisms culminate in the regulation of transcription to provide the necessary molecular adaptation. In the *Streptomyces* genus, the main mechanisms that orchestrate external signal-sensing and amplification include the autoregulatory DNA-binding transcription factors^7^, two-component systems^8^ and the extracytoplasmic function (ECF) sigma factors^9^. In ECF-dependent systems, the sigma factor directly controls gene expression by recruitment of the RNA polymerase (RNAP) to specific promoters. Control is mediated via the binding of the ECF-sigma by its cognate anti-sigma factor, usually coded by a gene that is co-transcribed with the ECF-sigma. In response to environmental stimuli, the anti-sigma releases the ECF-sigma, allowing the cell to moderate gene expression and respond to environmental fluctuations (reviewed in Lonetto, et al.^10^).

ECFs form the largest family of sigma factors, the group IV, which derive from *Escherichia coli* σ^70^. ECFs structure displays the minimal architecture required to bind DNA, the sigma σ^70^_region 2 (σ_2_) and σ^70^_ region 4 (σ_4_) conserved domains, which bind to the -10 and -35 promoter regions, respectively^11,12^. Canonical ECFs recognise conserved motifs in the -10 and the -35 boxes, often separated by an optimal spacer of 16-17bp^11^ to accommodate the binding of σ_2_ and σ_4_ to the promoters. The size of the linker region between σ2 and σ4 has been described to have an active regulatory role in both the recognition of the bipartite sequence motif and the binding to the RNAP^13,14^. The residues within the ECF σ2-σ4 linker are important for maintaining the bond between σ2 and σ4 and stabilize the binding of these regions to the RNAP. However, they do not seem to play a direct role in RNAP binding^14–16^.

Despite the discovery of ECFs in 1994^10^, detailed information regarding how these proteins evolved is still sparse. ECFs show great diversity and have been classified in 157 well-defined phylogenetic families^17–20^. In some of these families, ECFs harbour long extensions at the N and/or C terminal (NT and CT) encoding other conserved domains, besides the σ_2_ and σ_4_ DNA-binding domains^18,19^. A number of ECFs with CT extensions have been experimentally characterised (reviewed in Pinto et al.^21^), and in three of them the CT has been shown to perform a self-regulatory role that has replaced the anti-σ inhibitory function^22,23^. The best studied case is the ECF44 CorE from *Myxococcus xanthus* that harbours a CT cysteine rich domain (CRD), which promotes a compact conformation that is favourable for DNA binding^24,25^. ECF41 (σ^ECF41^) from *Bacillus licheniformis* is rendered inactive by the CT NTF2-like domain (IPR032710). A compact conformation of the protein is endorsed by a distal consensus motif (NPDKL) that links the SnoaL_2 domain to a signature motif in the σ2–σ4 linker (YVGPWLPEP). However, reducing the protein to its ECF core is not sufficient for effective transcription^22^. *Mycobacterium tuberculosis* sigma J (σ^J^_*Mtub*_) is another ECF in family ECF41 with a long CT extension that encodes the SnoaL-like domain (IPR037401, PF12680: SnoaL_2). The SnoaL_2 domain is an extension with approximately 100 residues that is a member of the NTF2-like superfamily. It shares identity with other conserved domains in this family such as SnoaL (PF07366) domain, the NTF2 domain (PF02136), or the PHZA_PHZB domain (PF03284). The SnoaL2 protein, harbouring the SnoaL_2 domain, was characterised as a hydroxylase involved in the biosynthesis of the antibiotic nogalamycin in *S. nogalacter*^26,27^. Both Direct Coupling Analyses (DCA) and the determination of the σ^J^_*Mtub*_ crystal structure have shown that the SnoaL_2 is a flexible region of the protein where its surface is tethered to the loop that links σ2 to σ4, to promote a compact conformation^23,28^. Although SnoaL_2 residues contact the ECF core^23^ and partial truncation of the SnoaL_2 domain led to an hyperactive sigma activity^22^, the full deletion of the CT domain abolished sigma factor activity, indicating that the CT extension does not serve as a σ^J^_*Mtub*_ antagonist^28^. It was suggested that the compact state could be a promoter-recognizing conformation, in which SnoaL_2—although not binding directly to the DNA—could stabilize the ECF binding to promoter, acting as a modulator of its activity^28^. A similar observation has been made for *B. licheniformis* ECF42 where the tetratricopeptide repeat (TPR) CT extension is necessary for its activity^29^. Even though the information collected suggests that, where present, SnoaL-like domains are important in regulating ECF-sigma activity, the physiological role of this extension remains unknown.

*Streptomyces tsukubaensis* NRRL18488 naturally produces FK506, also known as tacrolimus, which is frequently used as an immunosuppressant in clinical practice^30–32^. In *S. tsukubaensis,* stress-responsive transcription is coordinated by 42 ECFs, showing a level of investment in signalling that may reflect novel mechanisms of ECF-dependent transduction. Of the total ECFs, 8 harbour CT extensions in addition to the ECF core σ_2_ and σ_4_ conserved domains. In this work, we provide a functional characterisation of an ECF that contains a SnoaL_2 CT extension, SigG1. Although SigG1 might regulate itself through the SnoaL_2 domain, we identify RsfG as its cognate anti-sigma factor. Finally, we identify genes under SigG1 control and hypothesize how SigG1 orchestrates the timely progression of morphogenesis and secondary metabolism.

## Results

### *sigG1* encodes an ECF sigma factor with a SnoaL_2 extension

**Figure 1:**
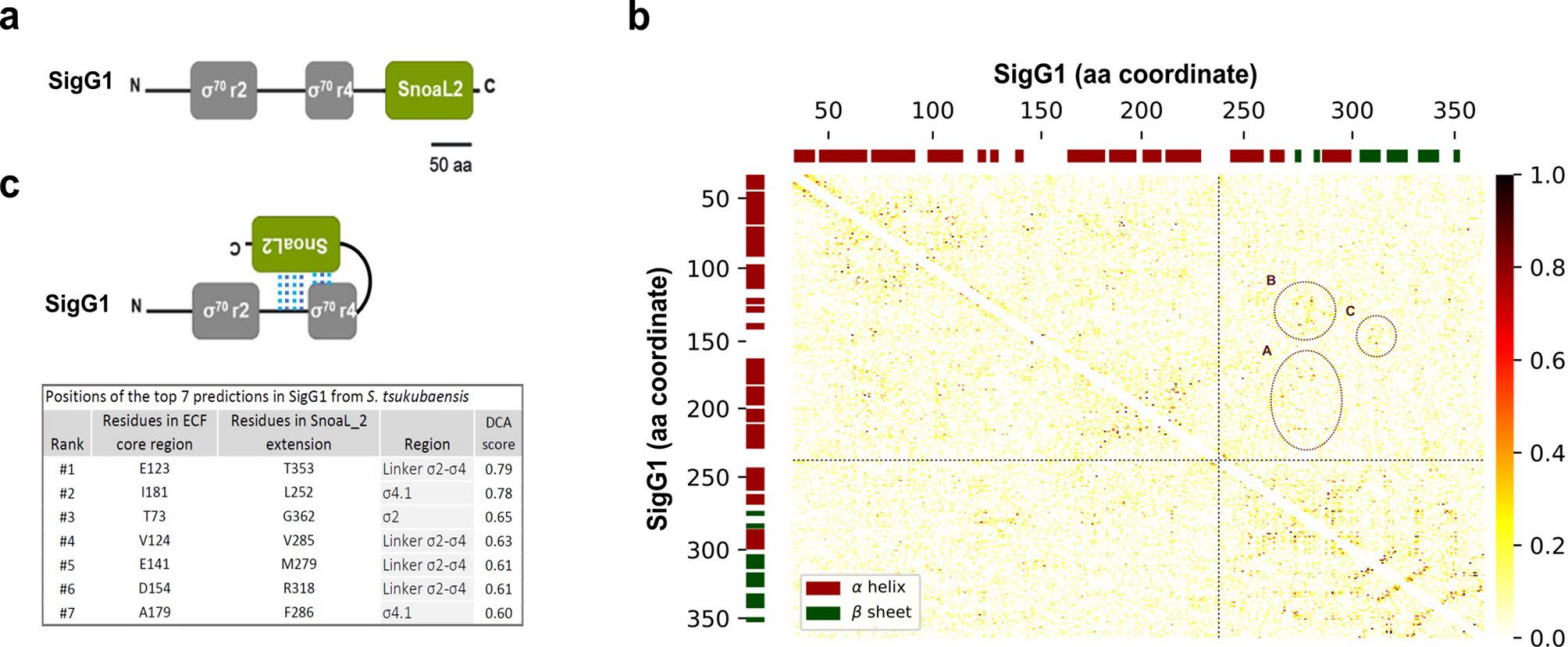
Predicted intramolecular interactions for ECF56. (**a**) Schematic representation of the Pfam domain organisation of the *sigG1* encoded protein. SigG1 harbours the σ2 (PF04542) and σ4 (PF04545) conserved domains, and an additional long C-terminal extension, coding for a SnoaL_2 domain (PF12680). (**b**) Contact map for predicted contacts between ECF core regions and the SnoaL_2 extension, using Direct Coupling Analyses (DCA). Each axis corresponds to the amino acids of SigG1 and the heatmap represents the DCA output, where darker spots correspond to higher scores. Scores > 1 are set to 1 in order to allow for the observation of smaller scores. Dashed lines split the core ECF regions from the C-terminal extension. Significant contacts between SnoaL_2 and core ECF regions are indicated by circles. (**c**) Model for compact conformation of SigG1 as predicted by DCA. DCA rank and scores of the significant contacts between ECF core regions and the SnoaL_2 domain are shown.

A gene encoding an ECF, *STSU_11560*, was identified in *S. tsukubaensis* located upstream of the gene that codes for OxyR—the major bacterial regulator of H_2_O_2_-induced stress response^33^. STSU_11560 harbours the amino terminal σ^70^_r_2_ (PF04542, σ_2_) and σ^70^_r_4_ (PF04545, σ_4_) conserved domains, and a C-terminal (CT) extension, encoding a SnoaL_2 domain (PF12680), which makes it distinctive from other typical ECF σ factors (Fig. 1a). Domains σ_2_ and σ_4_ are separated by an unusually long (53 aa) spacer. The presence of the CT SnoaL_2 domain in ECF sigma factors is distributed among different phyla^17,18^. They are predominantly found in the Actinobacteria and Firmicutes and, in particular, they are included within groups ECF41, ECF56, ECF205, ECF294 and ECF295 of the general ECF group classification (Supplementary Fig. S1)^17^. Besides STSU_11560, we identified three additional ECFs with a SnoaL_2 extension in the *S. tsukubaensis* genome: STSU_14518 and STSU_17474*,* that belong to the ECF41 group; and STSU_12530, a closer homologue to STSU_11560. These last two ECFs are enclosed within group ECF56, which is exclusively composed of ECFs with SnoaL_2 domains at the CT extension. STSU_11560 belongs to subgroup 3 of the ECF56 family (Supplementary Fig. S1).

The pool of SnoaL_2-containing ECFs in *S. tsukubaensis* resembles the organisation of orthologous ECFs experimentally characterised in the *Mycobacterium* genus. Sequence homology revealed that the full-length proteins encoded by *STSU_11560* and *STSU_12530* are similar to SigG from *Mycobacterium tuberculosis* H37Rv (40% and 47% identity, respectively), while STSU_14518 and STSU_17474 showed identity to SigJ from *M. tuberculosis* H37Rv (43% and 38% identity, respectively). The closest homologous protein to STSU_11560 found in the *Streptomyces* genus was a sigma-70 family RNA polymerase sigma factor from *Streptomyces katsurahamanus* (75% identity, E value: 2e^-168^). Following these analyses, we named the gene *STSU_11560* as *sigG1* and *STSU_12530* as *sigG2*. This work focuses on the physiological characterisation of *sigG1.*

### Direct coupling analyses (DCA) predict intramolecular contacts between SigG1 ECF core domains and SnoaL_2

ECFs with additional domains have been proposed to promote intramolecular contacts that regulate ECF activity^24,28,29^. Making use of the statistical method called direct-coupling analysis (DCA) we assessed whether the SnoaL_2 domain is folded towards the ECF domains σ_2_ and σ_4_. DCA is based on the covariation observed between pairs of residues that interact in large families of homologous proteins. When two residues interact, mutations in one of them need to be compensated by mutations in the second in order to preserve the contact. DCA is able to find pairs of amino acids that covariate due to their direct interaction^34^. DCA has been successfully used for the prediction of contacts between the core ECF and the SnoaL-like CT of ECF41 and the TPR-containing CT of ECF42, which later were proven to have an important regulatory role on ECF activity^23^. Here we found that DCA predicted a SigG1 tertiary structure that suggests contacts between the first half of SnoaL_2 CT and the linker that separates σ_2_ and σ_4_ (Fig. 1b, areas B and C). Additionally, DCA revealed a high probability of contacts between the N-terminal part of SnoaL_2 and the σ_4_ region (Fig. 1b, area A). These results suggest a potential self-regulatory function for SnoaL_2 (Fig. 1c).

### SigG1 interacts with a putative anti-sigma factor, RsfG

**Figure 2:**
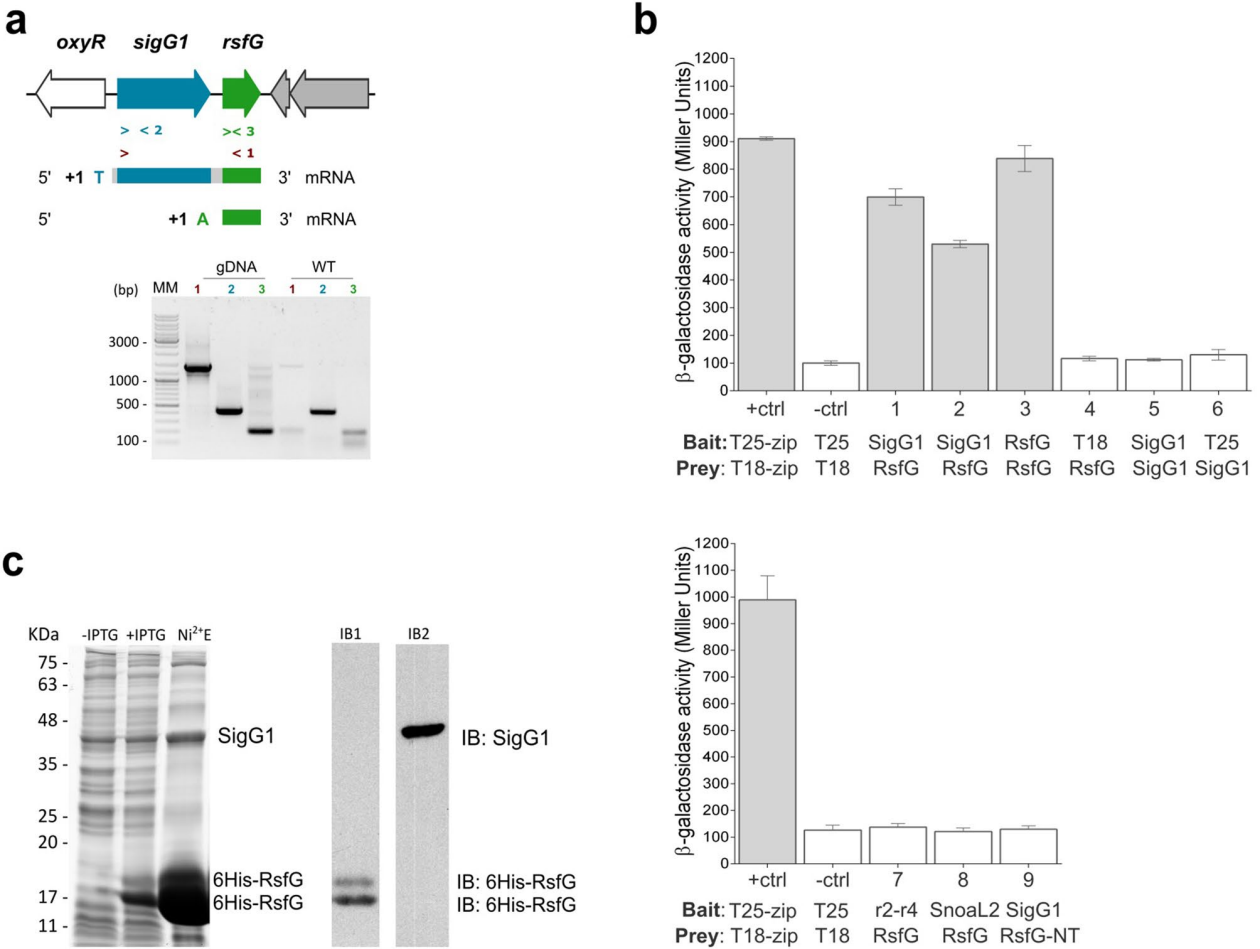
SigG1 interaction with RsfG (**a**) Co-transcription of *sigG1* and *rsfG* (lane 1) was observed by RT-PCR using as template DNase I-treated RNA from the wild-type (WT). Genomic DNA (gDNA) was used as PCR positive control. Transcription start sites (TSS) were mapped by 5′RACE PCR and sequencing. (**b**) BACTH assays for SigG1-RsfG binding in vivo. Positive controls pUT18Czip and pKT25-zip (+ ctrl); Negative control with empty vectors pUT18C and pKT25 (-ctrl); T18-SigG1 and T25-RsfG (1) SigG1-T18 and T25-RsfG (2); T18-RsfG and T25-RsfG (3); pUT18 and T25-RsfG (4); T18-SigG1 and T25-SigG1 (5); pUT18 and T25-SigG1 (6); T25-RsfG and truncated forms of SigG1 – T18-SigG1_r2-r4 region (7), T18-SigG1_SnoaL_2 domain (8); T25-RsfG_N-terminal and T18-SigG1 (9). Results are the average of three independent experiments. Grey bars indicate positive interaction between bait and prey. (**c**) Co-expression and co-purification of SigG1 and RsfG. SigG1 bound to the 6His-RsfG was eluted with 250 mM imidazole (Ni^2+^E). Immunoblot detection using an antibody against the His-tag (IB1) and a polyclonal antibody anti-SigG1 (IB2). Original uncropped images are presented in Supplementary Fig. S10.

Downstream from *sigG1* we identified an additional uncharacterised gene, *STSU_11555,* that encodes an hypothetical protein with no conserved Pfam domains. Many known ECFs are bound by their respective cognate anti-sigma factor (ASF), an interaction that renders the ECF inactive until it is released to direct transcription from stress-responsive promoters. The anti-sigma is usually encoded by a gene adjacent to the *ecf* and can be transcribed independently or co-transcribed in a single mRNA like for example, the *bldN*-*rsbN* pair in *S. venezuelae*^35^ or the *sigI*-*rsgI* pair in *Bacillus subtillis*^36^. To investigate whether *sigG1* and *STSU_11555* are co-transcribed, we assessed the transcription of both genes by RT-PCR using primers pairs that amplified at different locations in the mRNA. The RT-PCR revealed a longer transcript that included both sequences, indicating that *sigG1 *and *STSU_11555* are co-transcribed (Fig. 2a). The transcriptional start site (TSS) of *sigG1* was mapped by 5′ RACE 118 nucleotides upstream of the annotated start codon. This result was confirmed by analysis of existing RNA sequencing (RNA-seq) data^37^. In addition, we identified a TSS for *STSU_11555*, 74 nucleotides upstream of its coding region, indicating that it can also be transcribed from its own dedicated promoter, independently of *sigG1.*

To assess if SigG1 could be regulated posttranslationally by interacting with STSU_11555 we performed bacterial adenylate cyclase two-hybrid experiments (BACTH) and found that the two proteins physically interact strongly in *E. coli* (Fig. 2b). These results support the hypothesis that STSU_11555 could act as the SigG1 cognate anti-sigma factor. We therefore named this gene *rsfG* (regulator of sigma factor G1). Additionally, BACTH results show dimerization of RsfG that might function in homomultimeric complexes. Truncated versions of SigG1 and RsfG—where the σ_2_–σ_4_ region and the SnoaL_2 domain were individually tested against the full length RsfG; and the RsfG N-terminal part was tested against the full length SigG1—were not sufficient to promote interaction between the two proteins in the BACTH experiments, possibly because specific residues required for complex assembly were missing, or due to the loss of stable conformations of the proteins.

The formation of a SigG1-RsfG complex was confirmed via co-expression of the two coding sequences in *E. coli* using the pRSFDuet™-1 system. The coding sequences for SigG1 and RsfG were cloned into the IPTG-inducible vector to co-produce RsfG with an amino terminal (NT) histidine tag (6His-RsfG) and an untagged version of SigG1 in *E. coli*. By immobilizing the 6His-RsfG on a nickel agarose affinity resin (Ni-NTA) it was possible to pull-down the untagged sigma factor, indicating that RsfG and SigG1 form a complex (Fig. 2c). Elution of the purified proteins revealed two isoforms of 6His-RsfG, as identified by Peptide Mass Fingerprinting (PMF) and Western blot: one protein with the predicted 17 kDa and a second isoform with approximately 19 kDa. Size exclusion chromatography displayed a single peak corresponding to the SigG1/RsfG complex (Supplementary Fig. S2)).

### SigG1 binds to target promoters in vivo

**Figure 3:**
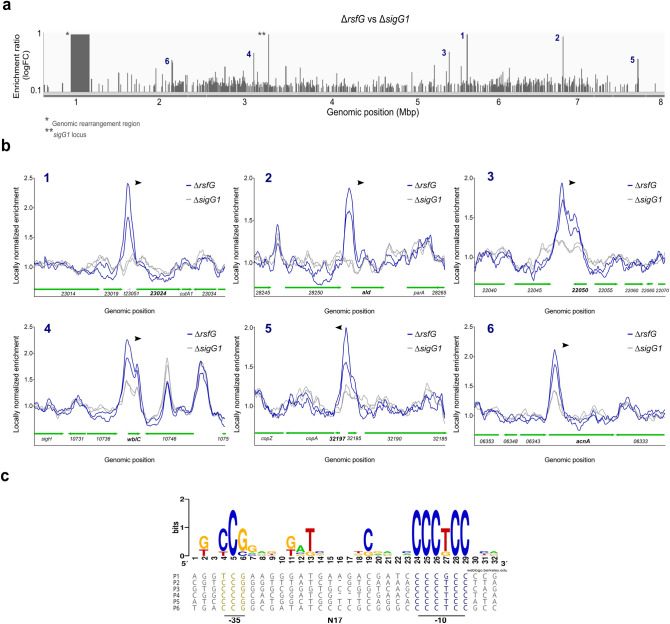
SigG1 binding to target promoters in vivo and in vitro (**a**) Genome-wide distribution of SigG1 binding sites identified by ChIP-seq analyses using the anti-SigG1 polyclonal antibody. Peak calling was performed from two independent biological replicates. (**b**) Close-up of a 2 kb region around the target genes. *S. tsukubaensis* Δ*rsfG:* blue. Negative control (Δ*sigG1*): grey. Genes in each genomic context are depicted in green (**c**) MEME consensus for SigG1 DNA-binding motif obtained by aligning the *sigG1*-dependent ChIP-seq enriched sequences with the MUSCLE algorithm. The consensus logo was obtained using the WebLogo platform^81^. P1: *STSU_23024p*; P2: *aldp*; P3: *STSU_22050*; P4: *wblCp*; P5: *STSU_32197p*; P6: *acnAp.*

To identify the set of genes under the direct control of SigG1 we generated a polyclonal antibody against SigG1 (α-SigG1) to be used in Chromatin Immunoprecipitation and Sequencing (ChIP-seq). Prior to ChIP-seq, we first replaced the *sigG1* and *rsfG* coding regions with an apramycin resistance cassette and generated single deletion mutants for these genes (Δ*sigG1* and Δ*rsfG*, respectively). The ChIP-seq experiments were performed in the *rsfG* null mutant to ensure SigG1 was available (i.e. not bound to the anti-sigma) to allow detection when bound to its target promoters. Analysis of *sigG1* transcript levels by RT-qPCR confirmed expression of *sigG1* in the *rsfG* mutant, and SigG1 protein was similarly detected by automated western blot (WES Simple) (Supplementary Fig. S3) confirming that the polyclonal antibody was suitable for use in ChIP-seq experiments. The *sigG1* null mutant was used as a negative control. DNA purified following immunoprecipitation using α-SigG1 was sequenced. The genomic regions with a fold-change enrichment of at least 1.5 (Δ*rsfG* vs Δ*sigG1*) were considered significant for peak calling. Using this approach we identified six peaks dispersed throughout the genome (Fig. 3a; Table 1).

**Table 1:** SigG1-binding regions identified by ChIP-seq analysis. FC – fold change.

Peak	Upstream gene ID	Downstream gene ID	Product	Location in genome	Distance (bp)*	Δ*rsfG/*Δ*sigG1* (LogFC)	*sigG1*-dependent**
1	*STSU_t23051*	*STSU_23024*	MFS transporter	2,553,980–2,554,012	44	1.04	N
2	*STSU_28250*	*ald* (*STSU_28255*)	Alanine dehydrogenase, Ald1	1,133,124–1,133,156	30	0.97	Y
3	*STSU_22045*	*STSU_22050*	Putative antisense RNA	2,781,241–2,781,273	na	0.73	Y
4	*STSU_10736*	*wblC* (*STSU_10741*)	WhiB-like transcriptional regulator, WblC	5,272,304–5,272,336	117	0.71	Y
5	*STSU_32195*	*STSU_32197*	Hypothetical protein	375,398–375,430	187	0.62	Y
6	*STSU_06343*	*acnA* (*STSU_06338*)	Aconitate hydratase, AcnA	6,313,866–375,430	1002	0.61	N

*Distance to START codon.

**Causal relationship upon *sigG1* deletion, RNA-seq.

Consistent with its function as a sigma factor, with the exception of peak 3, the remaining peaks are located in promoter regions (Fig. 3b). Strikingly, no clear peak was identified near the *sigG1* promoter suggesting that this sigma factor might not be subject to autoregulation via a positive feedback loop, a common feature of ECFs. SigG1 binds at the promoters of two genes related with morphological differentiation in streptomycetes*—*alanine dehydrogenase, *ald*^38^ and the WhiB-like regulator C, *wblC*^39^. We also identified three additional SigG1 target genes involved in metal-ion dependent homeostasis, *STSU_23024*, *STSU_32197* and aconitate hydratase, *acn*. Furthermore, peak 3 is located in the intergenic region between *STSU_22045* and *STSU_22050* with no apparent promoter region (Fig. 3b).

To investigate which genes were dependent on *sigG1* regulation, we assessed genome-wide transcription by total RNA sequencing (RNA-seq). We compared transcript levels in the wild-type and in Δ*sigG1* by analysing gene expression in MGm-2.5 liquid cultures at mid-exponential phase of growth—72 h (Supplementary Fig. S5). Gene expression results were validated by RT-qPCR on a selection of candidate genes (Supplementary Fig. S6) and the pattern of expression correlated with the one observed in the RNA-seq data. The level of transcripts for *hrdB* and *rspP* genes, encoding the primary essential sigma factor (HrdB) and the 30S ribosomal protein S16 (RspP) respectively, was monitored as an internal normalization control. A combination of the data obtained from the RNA-seq and the ChIP-seq experiments indicates that at least four target genes are members of the SigG1 regulon (Table 1). In addition, a careful inspection of RNA-seq data allowed to identify a putative promoter region for peak 3 identified in the ChIP-seq experiment. The data suggests that SigG1 binds to the upstream region of a putative antisense RNA of unknown function at the *STSU_22050* locus (Supplementary Fig. S4A). Surprisingly, inspection of both ChIP-seq and RNA-seq NGS data unveiled an unexpected genomic rearrangement in the Δ*sigG1* mutant. Genomic instability resulting in excision of large fragments of DNA has previously been described in the *Streptomyces* genus and other bacteria^40–44^. Through NGS re-sequencing of the full genome of this strain, we confirmed a 228 kb deletion in the terminal region of the chromosome from position 349,601 to 577,663 bp (Fig. 3a). No deletion was observed in the ∆*rsfG* strain.

Considering the central core of the ChIP-seq peaks, and the identification of the TSSs for each gene^37^, we were able to isolate and align promoter sequences harbouring the six identified peaks. By inputting the sequences for the *sigG1*-dependent genes into the MEME Suite tool^45^, we identified a putative consensus sequence for SigG1 DNA-binding, defined by the conserved residues CCG in the -35 element and the residues CCCTCC in the -10 element (Fig. 3c), separated by 17 bp non-conserved residues. The spacer size and the -10 box are in accordance with what was previously proposed for ECFs in the ECF56 group families^18^.

### *sigG1* is important for progression of morphological differentiation

**Figure 4:**
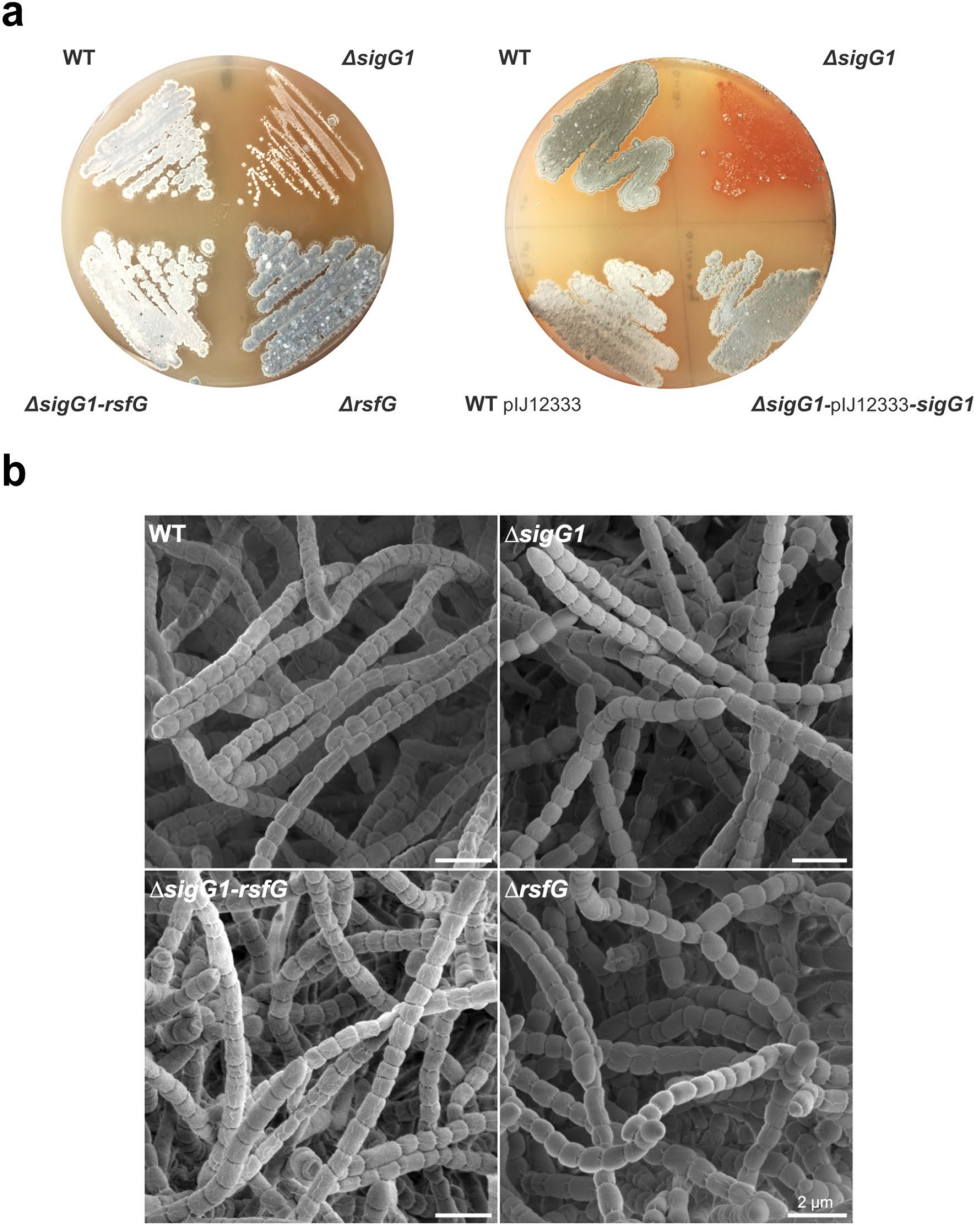
Phenotypes of the *S. tsukubaensis* wild-type, *sigG1* and *rsfG null* mutants. (**a**) 10^6^ spores were streak on ISP4 and grown for 14 days. To evaluate the phenotype of the complemented strains, 10^4^ spores were plated on ISP4. The pIJ12333 vector was used to express *sigG1* with its own promoter. The WT expressing the empty pIJ12333 is depicted as a control. (**b**) Differentiation into spores chains was examined by scanning electron microscopy (SEM), after 14 days of growth on ISP4. Scale bar: 2 µm.

To validate the *sigG1*-dependent recruitment of RNAP to the target promoters we have functionally characterised the deletion mutants for the newly identified ECF/ASF pair. One of the most significant peaks obtained with ChIP-seq was found in the promoter region of *ald* that codes for an alanine dehydrogenase enzyme, Ald1. Ald1 converts L-alanine residues, generated by protein turnover, into extra pools of pyruvate that help fuel metabolism for growth progression in *B. subtilis*^38^. Moreover, Ald1 is required for maturation of spores in *S. coelicolor*^46^. On ISP4 media, the *sigG1* null mutant exhibited impaired morphological development of sporogenic hyphae. After 6 days of growth on ISP4 agar, the wild-type strain had sporulated, whereas Δ*sigG1* lacked the spore-associated pigmented appearance of the wild-type (Fig. 4a). This phenotype was fully restored on agar plates by the introduction of a single copy of the *sigG1* wild-type allele under the control of its native promoter expressed from the ɸC31 integration site, located within *STSU_17107* in *S. tsukubaensis* (integration was confirmed by PCR). The delay in differentiation was further studied by scanning electron microscopy (SEM). SEM revealed that despite their initial white appearance, colonies of the *sigG1* mutant eventually fully differentiate leading to long chains of spores (Fig. 4b). We conclude that SigG1 is required for the timely differentiation of *S. tsukubaensis*. Strikingly, Δ*rsfG* grown on solid media exhibited a darker pigmentation that is usually associated with spores, whereas a double mutant for both *sigG1* and *rsfG* exhibited a partial reversal of the phenotype, as it resembled the wild-type morphology. Although some influence of the genomic gap in Δ*sigG1* strain would be expected, the phenotypic rescue observed in the complemented strain (that also contains the genomic deletion) shows that the morphological phenotype was mostly due to the absence of SigG1.

Using the RNA-seq data, we evaluated transcription in the Δ*rsfG* mutant and compared it to transcript levels in the wild-type. The identification of 18 significantly deregulated genes (Table 2; Supplementary Fig. S5; FDR < 0.05) revealed that *rsfG* plays a targeted role in the cell that is confined to the regulation of small operons. These encode for instance different types of proteases or, more interestingly, an operon encoding a putative ECF/ASF system of unknown function. Moreover, Pre-ranked Gene Set Enrichment Analysis (GSEA^47^) of Δ*rsfG* mRNA-seq profile showed an activation of genes involved in the TCA cycle (Supplementary Fig. S7).

**Table 2:** *rsfG*-dependent genes identified by RNA-seq analysis (FDR < 0.05). Volcano plot available in Supplementary Fig. S5

Gene ID	Product	Δ*rsfG/*WT (logFC)	*pFDR*
*STSU_03649*	Hypothetical protein	− 3.3	1.2E−02
*STSU_03654*	Trypsin-like serine protease	− 5.6	2.3E−04
*STSU_11545*	Hypothetical protein	1.8	2.6E−02
*STSU_11550*	Hypothetical protein	4.3	3.0E−04
*STSU_11560*	RNA polymerase factor sigma-70, SigG1	3.8	3.2E−03
*STSU_12320*	Subtilisin-like serine protease	− 3.2	1.5E−02
*STSU_16452*	Amino acid permease-associated protein	− 2.0	2.6E−02
*STSU_16977*	Hypothetical protein	− 2.9	1.4E−02
*STSU_16987*	Hypothetical protein	− 6.7	4.0E−05
*STSU_19470*	Transpeptidase	− 2.1	2.5E−02
*STSU_19475*	Hypothetical protein	− 4.9	2.9E−04
*STSU_19480*	ECF subfamily protein RNA polymerase sigma-24 subunit	− 6.5	4.0E−05
*STSU_25317*	Phospholipase	− 8.9	4.0E−05
*STSU_25322*	Protein phosphatase	− 6.7	2.7E−04
*STSU_25327*	Hypothetical protein	− 2.6	3.9E−03
*STSU_31170*	ErfK/YbiS/YcfS/YnhG family protein	− 8.8	2.7E−04
*STSU_31175*	Cytochrome P450 family protein	− 6.2	2.7E−04
*STSU_32005*	Rifamycin polyketide synthase, FkbB	− 1.8	2.1E−02

*FDR* false discovery rate, *FC* fold change.

### sigG1 is required for the maintenance of metal-ion homeostasis

**Figure 5:**
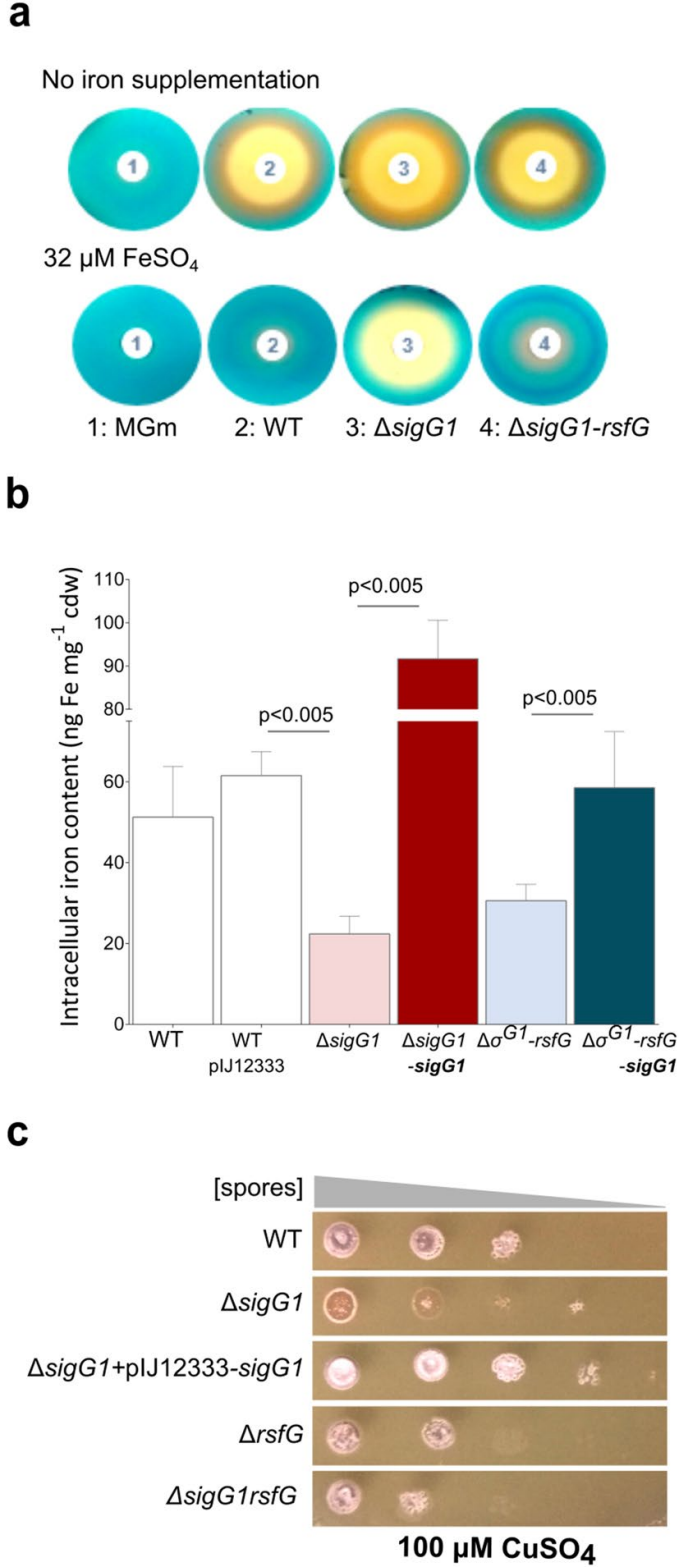
Evaluation of metal-ion homeostasis in *sigG1 mutants*. (**a**) Chrome azurol S (CAS) assay to determine siderophore secretion in the supernatants of strains grown in iron limiting and iron-sufficient conditions (FeSO_4_ 32 μM) conditions. (**b**) Intracellular iron content in *S. tsukubaensis* wild-type, Δ*sigG1*, Δ*rsfG*-*sigG1* and respective complemented strains measured by flame atomic spectrometry (F-AAS). Values are representative of at least three independent experiments. (**c**) Phenotypes of the wild-type, *sigG1* and *rsfG-related* strains grown on solid ISP4 supplemented with 100 µM CuSO_4_ medium for 6 days. Serial dilutions used were 10^5^, 10^4^, 10^3^, 10^2^, 10^1^ spores. Statistically significant differences were determined by one-way ANOVA followed by a Dunnet’s test (GraphPad Prism).

The SigG1 regulon includes two genes whose products are involved in the maintenace of metal-ion availability. *STSU_23024* shares homology to genes that have been linked to metal-ion dependent drug efflux systems^48^. It encodes a Major Facilitator Superfamily (MFS) transporter with 76% identity to the multidrug resistance protein Bmr3_2 from *Streptomyces sp. AVP053U2. STSU_32197*, is a newly identified gene that had not been annotated before (Supplementary Fig. S4B). It codes for an hypothetical protein and is part of an operon encoding a copper transport system. Hence, and given the high identity shared by the product of *STSU_23024* with metal-ion dependent transporter systems, we further asked if metal-ion homeostasis was compromised in the *sigG1* mutants. Using antiSMASH 4.0^49^ we identified at least three siderophore biosynthetic clusters in *S. tsukubaensis* predicted to encode Fe-enterobactin (STSU_33190- STSU_33135 FecCD), Fe-desferrioxamine B (STSU_23636- STSU_23676) and a second Fe-hydroxamate (STSU_16507- STSU_16442) siderophores. We assessed the ability of *S. tsukubaensis* to release siderophores, high affinity chelators that sequester ferric iron at very low concentrations to assist in metal-ion internalisation^50^. These small molecules are therefore secreted in iron-limiting conditions. Culture supernatants from cells grown in iron-limited conditions exhibited substantially higher levels of siderophores when *sigG1* was absent (as shown by the CAS assay in Fig. 5a) and, more strikingly, Δ*sigG1* produced siderophores in iron-replete cultures, revealing that these bacteria were under iron starvation. These results were corroborated by a 30% decrease in the intracellular iron content, as measured by flame atomic spectrometry (Fig. 5b). Noticeably, the intracellular iron content was restored upon complementation of the mutant strain (Fig. 5b), corroborating the importance of SigG1 in the maintenace of iron levels.

The emergence of a copper transporter system as a putative SigG1 target, prompted us to assess *sigG1*-dependent copper tolerance. Serial dilutions of spores of the wild-type, Δ*sigG1*, Δ*rsfG*, Δ*sigG1-rsfG* and the Δ*sigG1* strain complemented with *sigG1*, were inoculated on ISP4 copper-replete media (ISP4 supplemented with 100 µM CuSO_4_). After 6 days of growth, all *sigG1*/*rsfG*-related mutant strains exhibited an impaired growth when compared to the wild-type (Fig. 5c). Moreover, Δ*sigG1* spores were more sensitive towards copper than any of the other strains, in which spores fail to grow only in the highest dilutions tested, due to the toxicity of detrimetal concentrations of CuSO_4_. The dramatic arrest of development of aerial mycelium observed in Δ*sigG1* was reverted by expressing the *sigG1* wild-type allele in this mutant.

### The *S. tsukubaensis* oxidative stress response is dependent on *sigG1*

The disruption of metal ions homeostasis is frequently associated with concurrent impairment of the cell redox state. A major cause of toxicity is the cross-reaction of intracellular iron with H_2_O_2_—through the Fenton reaction^51^—that results in harmful levels of oxidative stress. To examine the impact of the fluctuations in iron availability upon *sigG1* deletion we assessed the maintenance of the redox balance by addressing the response to H_2_O_2_. The OxyR regulon is at the core of the bacterial defence against H_2_O_2_ stress. The location of *sigG1* relative to the *oxyR-ahpCD* operon in *S. tsukubaensis* supports the possibility that SigG1 has a role in the oxidative stress response.

**Figure 6:**
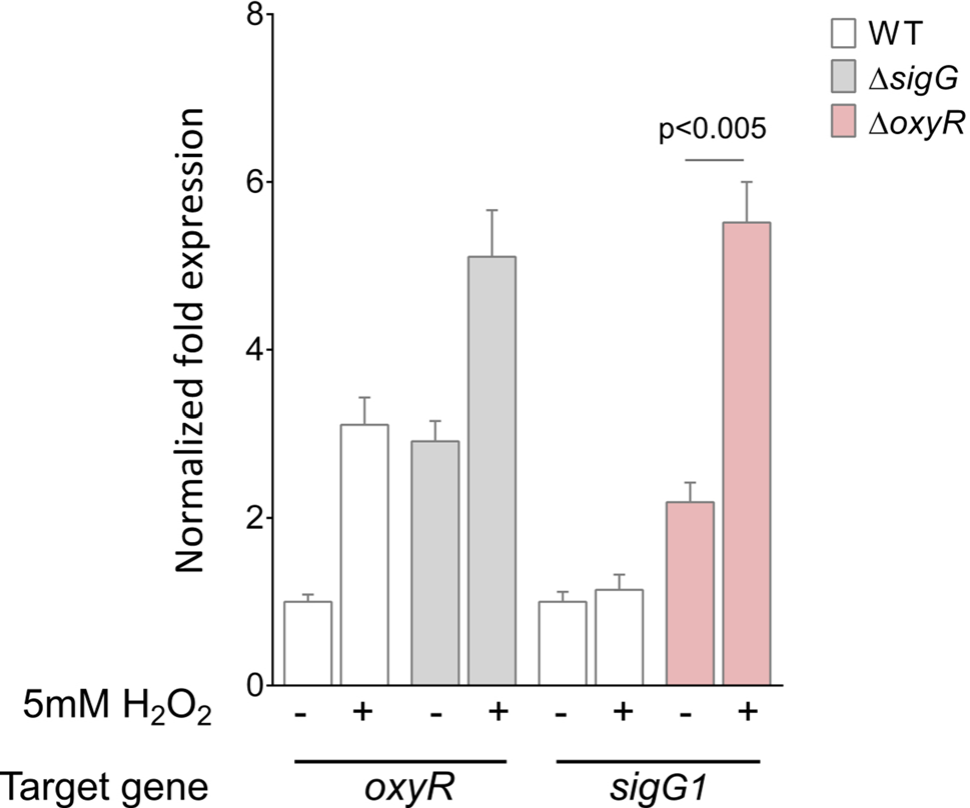
Stress-responsive gene expression. Transcription analyses of *oxyR* and *sigG1* expression in *S. tsukubaensis* mutant strains, upon a H_2_O_2_ stimulus evaluated by RT-qPCR. The Mean Normalised Fold Expression of the target genes was calculated relatively to the transcription of the reference genes (*rpsP* and *hrdB*) and internal normalisation was performed using the wild-type as the control. Results are representative of three biological replicates. Statistically significant differences to the wild-type were determined by one-way ANOVA followed by a Dunnet’s test (GraphPad Prism).

In order to investigate the role of SigG1 in the oxidative stress response, we exposed the cells to exogenous H_2_O_2_. After 15 min of H_2_O_2_ exposure, we examined *sigG1* transcript levels by RT-qPCR. Upon disruption of the major regulator of H_2_O_2_, *oxyR*, we observed an upregulation of *sigG1* transcripts in standard conditions, indicating that *sigG1* is expressed during the response to the oxidative stress damage (Fig. 6). Although transcription in the wild-type was not affected by the treatment, *sigG1 *levels were strongly induced by exogenous H_2_O_2_ in the absence of OxyR, indicating that *sigG1* is activated in response to H_2_O_2_-mediated oxidative stress. The transcript levels of *rsfG* were unchanged upon the ROS insult in these strains.

### Deletion of *sigG1* impairs secondary metabolism in *S. tsukubaensis*

Morphological differentiation and the oxidative stress response are tightly linked to secondary metabolism in the *Streptomyces* genus^52,53^. To evaluate if the morphological impairment observed on solid medium and the defective oxidative stress response displayed by ∆*sigG1* had an impact on secondary metabolism we assessed the production of FK506. In FK506-producing conditions (MGm-2.5 liquid cultures^54^), ∆*sigG1* showed no significant differences in growth when compared to the wild-type (Supplementary Fig. S8A) however we detected a delayed onset of FK506 biosynthesis, which resulted in a significant reduction (> 30%) in the total production when compared to the wild-type (Supplementary Fig. S8B). Nevertheless, we cannot exlude that the effect observed upon the production of FK506 could be influenced by the genomic deletion displayed by the ∆*sigG1* strain.

## Discussion

ECFs with additional domains have been predicted to rely on the autoregulatory activity of the interaction of the ECF conserved core with domains at the C-termini. SigG1 lacks the conserved NPDKL motif at the CT extension predicted to interact with the YVGPWLPEP motif in the linker between σ_2_ and σ_4_ of the σ^ECF41^ (Supplementary Fig. S9)^22,23^. In fact, DCA analysis predicted that SigG1 conformation is established by pairs of amino acid residues that are distinct from the ones that are in contact in σ^ECF41^. This suggests an alternative regulation of SigG1 in respect to ECF41. In addition, nearby *sigG1*, we found an adjacent gene encoding the RsfG protein, which was co-transcribed with *sigG1*. We observed that RsfG interacts with SigG1 acting as the cognate sigma antagonist to control SigG1 activity. Hence, we propose that intramolecular regulation in SigG1 might not be sufficient to fully inhibit transcription, as it requires an anti-sigma factor to prevent its runaway activity.

In previous studies in *S. natalensis*, we showed that the cell death of the vegetative mycelia that precedes the emergence of aerial mycelium results from an increase of the H_2_O_2_ intracellular pool^1^. Like in *S. coelicolor*^55^, in *S. tsukubaensis* the LysR regulator OxyR perceives the H_2_O_2_ signal and drives transcription of alkylhydroperoxidases to detoxify the excess of these reactive oxygen species (ROS) (Pires et al. unpublished data). In parallel, the OxyR regulon includes other players that maintain metal-ion homeostasis. In this study, we provide evidence that the antioxidant response in *S. tsukubaensis* harbours novel players that compose an ECF sigma/anti-sigma system. In the 147 *Streptomyces* genomes within ECF56 family in our database, we found a gene encoding an OxyR-like transcriptional regulator in the proximity of a SigG1 homologue-encoding gene. We hypothesize that SigG1 is likely to be an ECF that responds to H_2_O_2_ intracellular signals, generated by developmental transitions, to activate the cascades of genes that regulate the metabolic shift during growth. *M. tuberculosis* SigJ has also been implicated in the response to oxidative stress. Despite the susceptibility to H_2_O_2_ exhibited by a Δ*sigJ* mutant, *sigJ* mRNA levels are not responsive to H_2_O_2_-induced stress^56^.

The control of intracellular H_2_O_2_ homeostasis is linked with the homeostasis of metal-ion content as the cells face emergent toxicity due to the Fenton reaction^51^. Characterisation of ∆*sigG1* strain suggests that SigG1 participates in the control of fluctuations in iron and copper availability in *S. tsukubaensis*. Note that despite the genome gap displayed by the ∆*sigG1* strain, complementation with a copy of *sigG1* restored the wild-type phenotype, not only regarding intracellular iron and copper toxicity (Fig. 5) but also the morphological phenotype (Fig. 4). The SigG1 regulon includes genes encoding proteins with iron-sulphur centres that are known regulators of metal-ion homeostasis, including AcnA and WblC. In streptomycetes, WblC is involved in developmental cascades and, moreover, it regulates the expression of the gene encoding SigR, a well-studied sigma factor responsible for coordinating the response to oxidative stress induced by thiol agents^57^. Furthermore, SigG1 regulates the expression of two genes encoding putative metal-ion dependent efflux systems described in other bacteria. Together with a disrupted iron balance, we show that strains defective in *sigG1* were significantly more sensitive to exogenous copper than the wild-type. The mechanisms controlling copper uptake or secretion in *Streptomyces* species were recently identified^58–61^*.* The function of the product of *STSU_32197* remains unknown since no clear homologues were identified for this protein. However, one possibility is that SigG1 mediates copper tolerance by activating an operon for the coding sequences for the copper chaperone/P-type ATPase system CopA/CopZ (*STSU_32197-STSU_32205*) responsible for the secretion of these metal-ion in *S. lividans.* Similarly to ferrous iron, copper ions can also react with intracellular thiols to produce hydroxyl radicals (the Haber Weiss reaction). Moreover, by occupying a high position in the Irving-Williams series, copper can substitute iron as a cofactor for transcriptional regulators or enzyme activity^62^, which damages iron-sulphur centres due to displacement of the iron ions^63^. Our hypothesis is that to accompany the transcriptional activation of iron-sulphur clusters containing proteins, SigG1 activates the *copA/copZ* copper efflux locus to limit copper concentration in the cytoplasm and avoid toxicity. In the absence of *sigG1,* cells fail to export copper and to avoid an increase in oxidative stress, cells can trigger the export of iron to balance redox active metals in the cytoplasm. A low intracellular iron content prompts bacteria to recover environmental iron bound to siderophores, which in turn suppress copper-induced ROS toxicity^64^. Previous works have demonstrated that cuproproteins are recruited for the developmental switch from vegetative to aerial mycelium^60,65^, which is consistent with the timeframe of SigG1 activity. Overall, our results suggest that SigG1 might be involved in regulating iron and copper homeostasis, probably in an effort to counteract the metal-ion imbalance caused by oxidative damage.

Curiosly, no SigG1 binding site was identified upstream of *sigG1*. Instead, *sigG1* promoter harbours a conserved motif described for the sigma-factor BldN, a developmental determinant regulated by iron availability at the interface of vegetative to aerial growth^35,66^. The potential BldN-dependent regulation of *sigG1* could explain the iron limitation and the impaired aerial growth observed for *S. tsukubaensis* Δ*sigG1.*

Altogether, these findings provide an indication as to how *Streptomyces* could protect itself from oxidative stress generated endogenously at the colony core. We hypothesize that as the rate of primary metabolism slows down, nutrient depletion provides the oxidative trigger required to activate SigG1. SigG1 responds by activating the expression of Ald1, which catalyses the alanine from protein turnover, leading to increased pyruvate production during sporulation. AcnA will then promote pyruvate conversion into energy through the TCA cycle. This extra energy, together with a balance in intracellular metal-ion promoted by other SigG1 targets, will support the metabolic processes responsible for the developmental shift to aerial differentiation. The presence of a SigG1-dependent promoter upstream of the aconitate hydratase encoding gene and the upregulation of the genes that composed the TCA cycle molecular signature, observed when deleted *rsfG,* support this idea (Supplementary Fig. S7). Moreover, we observed the enhanced transcription of genes encoding other proteins that are directly involved in metal-ion homeostasis (*sodA*, *bfr*) in the Δ*rsfG* mutant (*p* > 0.05).

Transitions between mycelia differentiation stages are associated with the onset of secondary metabolism. Another important finding in our study using the industrial antibiotic producing conditions was that disrupting the sigma factor decreased the production of FK506 (Supplementary Fig. S8). This further reinforces SigG1 as an important regulator for the timely progression of growth in *S. tsukubaensis* and, by association, for the production of FK506. Although in the latter case we cannot completely exclude an indirect effect of the genomic deletion displayed by ∆*sigG1*.

To the best of our knowledge, this is the first work describing the physiological role of an ECF with a SnoaL_2 extension in bacteria. Overall, the insights gained in this work strengthens the link between morphological development, metal-ion homeostasis and oxidative stress in *Streptomyces tsukubaensis,* in which, via the activation of a unique SnoaL_2-containing ECF, *S. tsukubaensis* can respond to stress and thrive in the complex environment of the soil.

## Materials and methods

### Bacterial strains and growth conditions

Bacterial strains used in this study are described in Table 3. *Escherichia coli* strains were grown under aeration in LB (Lysogeny Broth) liquid medium or on LB agar at 30 °C or 37 °C. *Streptomyces tsukubaensis* strains were grown at 30 °C on Difco ISP4 solid medium (BD, NJ, USA) for spore production. Liquid cultures were grown in MGm-2.5 medium at 28 °C, under aeration at 220 rpm^54^. Ampicillin at 100 μg/mL, kanamycin 50 μg/mL, hygromycin 25 μg/mL, apramycin 50 μg/mL, chloramphenicol at 25 μg/mL or thiostreptone at 40 μg/mL where added to the media when required. Phenotypic analysis of *S. tsukubaensis* and derived strains was conducted on ISP4 solid media or in MGm-2.5 liquid cultures.

**Table 3:** Strains and plasmids used in this study.

Strains or plasmids	Description	Source/Reference
***S. tsukubaensis***		
NRRL 18488	Wild-type	^82^
Δ*sigG1*	Δ*sigG1*::*apr*; Apr^R^	This study
Δ*rsfG*	Δ*rsfG*::*apr*; Apr^R^	This study
Δ*sigG1-rsfG*	Δ *sigG1-rsfG*::apr; Apr^R^	This study
WT pIJ12333	Wild-type expressing pIJ12333 empty vector	This study
Δ*sigG1* pIJ12333 + *sigG1*	Δ*sigG1*::apr expressing pIJ12333-*sigG1*; Tsr^R^ Apr^R^ (ɸC31-transduction)	This study
Δ*sigG1-rsfG1* pIJ12333 + *sigG1*	Δ*STSU_11555-60*::apr expressing pIJ12333-*STSU_11560*; Tsr^R^ Apr^R^ (ɸC31-transduction)	This study
∆*oxyR*	Δ*oxyR::apr*; Apr^R^	(Pires et al., unpublished results)
***E. coli***		
BTH101	F- *cya-99 araD139 galE15 galK16 rpsL1 (Strr) hsdR2 mcrA1 mcrB1*	^75^
BW25113 [pIJ790]	Δ(*araD-araB*)*567*, Δ*lacZ4787*(::*rrnB-4*), *lacIp-4000*(*lacI*^*Q*^), λ-, *rpoS369*(Am), *rph-1*, Δ(*rhaD*-*rhaB*)*568*, *hsdR514*; Cm^R^	^83^
DH5α	F- ɸ80*lacZ*∆M15 ∆(*lacZYA-argF) U*169 *recA1 endA1 hsd*R17*(*rK-, mK +*) pho*A *sup*E44 λ*– thi-1 gyr*A96 *relA1*	
ET12567 [pUZ8002]	*dam, dcm, hsd*, Kan^R^ , Cm^R^	^84^
Nico21	*can*::CBD *fhuA2* [*lon*] *ompT gal* (λ DE3) [*dcm*] *arnA*::CBD *slyD*::CBD *glm*S6Ala ∆*hsdS* λ DE3 = λ *sBamHIo* ∆*EcoRI-B int*::(*lacI*::P*lac*UV5::T7 gene1) *i21* ∆*nin5*	New England Biolabs
**Plasmids**		
pIJ12333	Vector for conjugal transfer of DNA from *E. coli* to *Streptomyces* sp.; integrative (ɸC31-transduction), Hyg^R^ Tsr^R^	^85^
pIJ12333-*sigG1*	pIJ12333 carrying *sigG1* preceded by its native promoter, Tsr^R^	This study
pIJ773	Plasmid template for amplification of the *apr-oriT* cassette for Redirect™ PCR-targeting	^67^
pIJ790	Modified l RED recombination plasmid [oriR101] [repA101(ts)] araBp-gam-be-exo, Cm^R^	^67^
pUZ8002	RP4 derivative with defective oriT, Kan^R^	^84^
pET15b	T7 expression vector, Amp^R^	Novagen
pET15b-sigG1	pET15b-*sigG1* full length coding sequence	This study
pRSFDuet-1™	T7 co-expression vector. Contains two multiple cloning sites (MCS), Kan^R^	Novagen
pRSFDuet3	pRSFDuet-1 carrying *rsfG* cloned into MCS1 and *sigG1* cloned into MCS2 of pRSFDuet-1	This study
pUT18C	Two-hybrid vector, N-terminal cyAT18fusion, Amp^R^	^75^
pUT18	Two-hybrid vector, C-terminal cyAT18fusion, Amp^R^	^75^
pKT25	Two-hybrid vector, N-terminal cyAT25fusion, Kan^R^	^75^
pKNT25	Two-hybrid vector, C-terminal cyAT25fusion, Kan^R^	^75^
pKT25-zip	Derivative of pKT25 carrying the leucine zipper of GCN4 fused in frame to T25	^75^
pUT18C-zip	Derivative of pUT18C carrying the leucine zipper of GCN4 fused in frame to T18	^75^

### Construction of deletion mutant strains

In-frame deletions Δ*sigG1*::apr, Δ*rsfG*::apr and Δ*sigG1-rsfG*::apr were generated using REDIRECT PCR targeting technology^67^. Briefly, native *sigG1* was replaced with the *apr-oriT* cassette on the 15C1 cosmid using primers red_sigG1_F/Rand red_rsfG_F/R (Supplementary Table S1). Modified cosmid was transfer to *S. tsukubaensis* WT by intergeneric conjugation^68^ and apramycin resistant recombinants selected. In-frame deletions were confirmed by PCR (primers red_conf_sigG1_F/R and red_conf_rsfG_F/R; Supplementary Table S1) and by Southern hybridization. For complementation of the *sigG1* deletion mutant strain, *sigG1* and its promoter region were amplified using oligonucleotides SigG1_SpeI_F and SigG1_SpeI_R, cut with *SpeI* and cloned into the *XbaI* site of pIJ12333. The construct was introduced into *S. tsukubaensis* Δ*sigG1* by conjugation.

### Scanning electron microscopy

Samples for scanning electron microscopy were obtained by growing strains on ISP4 agar for 15 days and prepared as previously described^69^ with minor modifications. Isolated colonies were mounted on an aluminium stub using Tissue Tek (BDH Laboratory Supplies, Poole, England). The stub was then immediately plunged into liquid nitrogen slush at approximately − 210 °C to cryo-preserve the material. The frozen sample was transferred, in vacuo, onto the cryostage of an ALTO 2500 cryo-transfer system (Gatan, Oxford, England) attached to a FEI Nova NanoSEM 450 (FEI, Eindhoven, The Netherlands). Sublimation of surface frost was performed at − 95 °C for 3½ min before sputter coating the sample with platinum for 2½ min at 10 mA, at colder than − 110 °C. After sputter-coating, the sample was moved onto the cryo-stage in the main chamber of the microscope, held at − 125 °C, and imaged at 2.6 to 3 kV.

### *S. tsukubaensis* genomic DNA isolation

Genomic DNA from *S. tsukubaensis* strains was obtained with the Master Pure Gram-positive DNA Purification Kit (Epicentre, WI, USA) or with the optimized procedure for Gram-positive bacteria from the GeneJET Genomic DNA Purification Kit (Thermo Fisher Scientific, MA, USA) according to the protocol provided by the manufacturers. *S. tsukubaensis* genome pair-end resequencing was performed in an Illumina (NovaSeq 6000) platform using a 350 bp PCR-free library (Novogene, Hong Kong).

### Polymerase chain reaction (PCR) and oligonucleotides used

DNA fragments used in this study were obtained by PCR using the G2 GoTaq (Promega, WI, USA) or Q5 High-Fidelity DNA Polymerase (New England Biolabs, MA, USA). All oligonucleotides used are listed in Supplementary Table S1.

### RNA isolation and RT-qPCR analyses

Samples for gene expression studies were harvested at 72 h (mid-exponential phase), 96 h (late exponential phase) or 120 h (early stationary phase) of growth. For H_2_O_2_ induced stress experiments, samples were collected at 72 h (t_0_) and 15 min after the addition of 5 mM H_2_O_2_ (t_1_). Culture aliquots were mixed with two volumes of RNA Protect Bacteria Reagent (Qiagen, Hilden, Germany) and maintained for 5 min at room temperature. Cells were harvested by centrifugation and immediately frozen by immersion in liquid nitrogen. The total RNA was isolated using the RNeasy Mini kit (Qiagen) according to manufacturer instructions with modifications described in Beites, et al.^52^. RNA quality and integrity were evaluated in an Experion Automated Electrophoresis System (Bio-Rad, CA, USA). For cDNA synthesis, 1 μg of DNase I-treated total RNA was transcribed with the iScript Select cDNA Synthesis Super Mix Kit (Bio-Rad). RT-qPCR amplifications were performed in an iCycler iQ5 Real-Time PCR detection system (Bio-Rad) using 0.2 μM of each primer (Supplementary Table S1) and using, 10 μL of KAPA SYBR FAST RT-qPCR Master Mix (KAPA Biosystems, MA, USA) and 2 μL of template cDNA. Standard serial dilutions of the cDNA were used to check the relative efficiency and quality of each primer pair. Non-template controls were included. A melting curve analysis was performed at the end of each RT-qPCR to exclude the formation of nonspecific products. Analysis included three biological replicates and technical triplicates for each cDNA. The data obtained was analysed using the method described by Pfaffl^70^. For each analysis *rpsP* and *hrdB* mRNAs were used for normalization.

### Mapping of the 5′ terminus of mRNA by Rapid Amplification of cDNA Ends (5′ RACE)

Transcriptional start site (TSS) identification was performed using the 5′ RACE System for Rapid Amplification of cDNA Ends, Version 2.0 kit (Thermo Fisher Scientific), following the manufacturer’s instructions. First strand cDNA synthesis was carried out using the gene-specific primer GSP1 (Supplementary Table S1). PCR amplification of tailed cDNA was carried out using the 5’-RACE abridged anchor primer (AAP) with the GSP2 nested primer (Supplementary Table S1). Specificity of the PCR products was confirmed by re-amplification using the AUAP primer and a GSP3 nested primer, and by sequencing.

### RNA sequencing

For the genome-wide transcriptomics experiments, mycelia samples were harvested from liquid cultures at 72 h, and total RNA was isolated as described above. Quality control of the total RNA was assessed through the RNA integrity number (RIN). The library construction of cDNA molecules from total RNA samples was carried out using TruSeq Stranded Total RNA with Ribozero Library Preparation Kit (Illumina, CA, USA). The generated DNA fragments (DNA library) were sequenced in the lllumina Hiseq 4000 platform, using 150 bp paired-end sequencing reads (Stab-Vida, Portugal).

### Preparation of cell-free protein extracts

Cells were suspended in lysis buffer containing 50 mM potassium phosphate buffer (pH 6.8) supplemented with Complete EDTA-free protease inhibitor cocktail (Roche, Mannheim, Germany) and lysed on ice using a sonifier. Protein concentration was determined using the BCA Protein Assay Reagent (Thermo Fisher Scientific). Bovine serum albumin was used to determine standard curves.

### Secreted siderophores detection

Siderophore production was assessed using the Chrome Azurol S (CAS) assay^71^. Cultures were grown in MGm-2.5 media with or without ferrous iron supplementation. Samples were harvested at 96 h and supernatants placed on CAS agar plates at 30 °C.

### Determination of total iron content

Extracellular iron levels were determined in samples harvested throughout growth in MGm-2.5. Cells were centrifuged and the supernatant was recovered to determine total extracellular iron content, using the QuantiChrom Iron Assay Kit (BioAssay Systems, CA, USA) according to the manufacturer's instructions. Intracellular iron levels were measured by flame atomic absorption spectrometry (F-AAS) following a procedure adapted from Yang et al.^72^. Samples were collected from liquid cultures by centrifugation and cell pellets were washed three times with TE buffer (20 mM Tris–HCl, 5 mM EDTA, pH 7.7), followed by one wash with metal-free double distilled water to remove salts. Cells were suspended in 65% (v/v) HNO_3_, lysed at 75 °C and the supernatant was analysed for Fe content by F-AAS at 248.3 nm, using a PU 9200X spectrophotometer (Philips). Fe content was normalized to protein concentration.

### FK506 quantification

FK506 production by *S. tsukubaensis* strains was quantified by HPLC as previously described^73^ with minor modifications. Extraction of FK506 was carried out by mixing 1 mL of culture with 1 mL of 100% methanol, for 1 h at 30 °C. The mixture was centrifuged and the supernatant was recovered and analysed using a SunFire C18 column (4.6 × 150 mm, 3.5 μm; Waters, MA, USA) in a HPLC system (Hitachi, Tokyo, Japan). The UV detector was set at 210 nm and the oven was set at 55 °C. Elution was performed with a gradient mobile phase of 0.1% (v/v) trifluoroacetic acid (TFA) and 20% (v/v) Methyl tert-butyl ether (MTBE) in acetonitrile.

### Overexpression and purification of recombinant 6His-tagged SigG1 by IMAC

The full-length coding sequence of *sigG1* was cloned into the *Nde*I and *Xho*I restriction sites of the pET15b vector (Novagen) and protein overexpression was induced with 0.2 mM IPTG, at 16 °C, 180 rpm. The N-terminal hexa-histidine tagged SigG1 (6His-SigG1) was isolated using metal ion affinity chromatography (IMAC). Cells were recovered by centrifugation, resuspended in lysis buffer (20 mM sodium phosphate, 0.3 M NaCl, 10 µg/mL DnaseI, 200 µg/mL lysozyme, protease inhibitor cocktail pH 7.4) and lysed using a French press homogenizer at 8000 psi. After centrifugation, the clear extract was loaded onto a 1 mL HisTrap Niquel Sepharose High Performance column (GE healthcare, IL, USA) pre-charged with Ni^2+^ and equilibrated with binding buffer (20 mM sodium phosphate, 300 mM NaCl, 20 mM imidazole, pH 7.4). The column was attached to a BioLP Fast Protein Liquid Chromatography (FPLC) system (Bio-Rad) and further washed with binding buffer at a flow rate of 1 mL/min. Elution was carried out to an adequate level of purity with approximately 120 mM imidazole, and fractions were collected and analysed by SDS-PAGE. Peptide mass fingerprinting (PMF) confirmed the identity of the protein. Buffer exchange to 20 mM sodium phosphate, 300 mM NaCl was carried out. The protein was dialysed and concentrated using an Amicon Ultra-15 30Kda (Millipore, MA, USA).

### Production of SigG1-specific polyclonal IgY antibodies

The 6His-SigG1 purified protein and the V16-A39 epitope (SigG1^V16-A39^) were used to induce immunization of quail for the production of the anti-SigG1 polyclonal antibodies in egg yolks at HenBiotech (Coimbra, Portugal).

### Immunoblot detection of SigG1 and 6His-RsfG

Total protein extracts were run on 10% SDS-PAGE and transferred to a nitrocellulose membrane. Membranes were incubated with blocking buffer (5% (w/v) dried milk in TPBS/TBST), rinsed twice in TPBS/TBST and further incubated with blocking buffer supplemented with anti-SigG1 polyclonal quail antibody (final dilution 1:1000) or tetra-his mouse monoclonal antibody (Qiagen), final dilution 1:5000. After incubation, the membranes were rinsed twice in TPBS/TBST, and incubated with secondary antibody—rabbit anti-chicken IgY (IgG) coupled to peroxidase (A9046, Sigma-Aldrich, MI, USA) or anti-mouse IgG conjugated to HRP (Santa Cruz Biotechnology, CA, USA). Signals were revealed with Prime Plus ECL detection kit (Bio-Rad).

### Recombinant expression and purification of SigG1-RsfG complex

For the co-expression studies, the full-length coding sequences of *rsfG* and *sigG1* were cloned into the MCS-I (*EcoRI* and *HindIII* sites) and MCS-II (*NdeI* and *KpnI* sites), respectively, of the pRSFDuet-1 (Novagen) vector to generate a poly-histidine tag RsfG recombinant protein and a SigG1 untagged recombinant protein. Expression of soluble proteins in *E. coli* Nico 21 cells (New England Biolabs) was achieved after 20 h incubation with 1 mM IPTG, at room temperature, with aeration. The molecular weights of the recombinant proteins were verified by nanoLC–MS/MS. Cells were suspended in lysis buffer (20 mM sodium phosphate, 0.15 M NaCl, 10 µg/mL DNaseI, 200 µg/mL lysozyme, protease inhibitor cocktail pH 7.4) and disrupted by mechanical lysis through a FRENCH Press (Thermo Scientific) at 4000 psi. The soluble co-expressed proteins were batch purified in 0.15 M NaCl, 20 mM sodium phosphate buffer, pH 7.4 using a Ni-NTA agarose affinity chromatography matrix (Qiagen). Elution was performed in 250 mM imidazole and analysed by SDS-PAGE. The protein fractions were concentrated in 10 kDa cut-off Amicon filters. Analytical size exclusion chromatography (SEC) was used to probe the molecular weight of the SigG1-6His-RsfG complex using a Superose12 10/300 GL analytical grade column (GE Healthcare) connected to an ÄKTA Purifier 10 system (GE Healthcare). Protein was eluted in 20 mM sodium phosphate buffer containing 150 mM NaCl, pH 7.4. The experimental molecular weight of the complex was determined by plotting the elution volume against a standard curve with the following standards: RNase A (13.7 kDa), chymotrypsinogenA (25 kDa), ovalbumin (44 kDa) and BSA (66 kDa).

### Automated western blot (Wes)

Analysis of the native SigG1 protein in crude *S. tsukubaensis* extracts was performed using the quantitative Wes capillary electrophoresis and blotting system (ProteinSimple, CA, USA) with the Wes No Secondary Detection (12 to 230 kDa) Master kit. Protein samples were prepared in accordance with the manufacturer’s directions. Labelling was achieved using the anti-SigG1^V16-A39^ antibody and a rabbit anti-chicken IgY (IgG) coupled to peroxidase antibody (A9046, Sigma). Results were analysed using the Protein Simple software Compass (version 2.6.7).

### ChIP-sequencing

For the Chromatin Immunoprecipitation (ChIP) assays *S. tsukubaensis* strains were grown in MGm-2.5 and samples were harvested and prepared as described in Gallagher, et al.^74^ with the following modifications. For immunoprecipitation of SigG1 cross-linked DNA, the total extract was incubated overnight with polyclonal anti-SigG1^V16-A39^ antibody at 4 °C. Immunoprecipitation was carried out using a goat anti-chicken IgY agarose (ab76444, Abcam) for 4 h. Genomic DNA libraries enriched for SigG1 binding were produced from these samples, size selected to ~ 100–500 bp, and sequenced on an Illumina NovaSeq 6000 platform (Novogene, Hong Kong), using 150 bp paired-end reads. These experiments were performed in two biological replicates.

### Bacterial two-hybrid assays

Bacterial adenylate cyclase two-hybrid assays (BACTH, Euromedex, Souffelweyersheim, France) were performed as described previously^75^. Briefly, the DNA fragments that encode the full-length RsfG and SigG1 proteins were cloned into BACTH T18 and T25 containing vectors*.* The empty plasmids and the zip plasmids were used as negative control and positive control, respectively. Plasmids were co-transformed into *E. coli* BTH101 and incubated at 30 °C for 2–3 days. Transformants were grown on M63/MacConkey agar supplemented with 0.3% lactose, 0.5 mM Isopropyl-b-D-1-thiogalactopyranoside (IPTG), the β-galactosidase chromogenic indicator 5-bromo-4-chloro-3-indolyl-b-D-galacto-pyranoside (X-Gal, 40 µg/ml) and appropriate antibiotics, at 30 °C for 3–7 days*.* The positive clones were assessed using the β-galactosidase assay^76^. Single colonies of the co-transformed bacteria were expanded overnight in LB broth containing X-Gal at 100 mg/ml, 0.5 mM IPTG and antibiotics for selection. Cells were harvested and resuspended in Z buffer (60 mM Na_2_HPO_4_, 40 mM NaH_2_PO_4_, 10 mM KCl, 1 mM MgSO_4_, 50 mM b-mercaptoethanol pH 7.0). Cells were permeabilized by mixing with toluene. Permeabilized cells were mixed with 10 mg/ml ortho-nitrophenyl-β-galactoside (ONPG) and OD_405nm_ was measured over time. β-galactosidase activity units were calculated as previously described by Miller ^76^.

### Bioinformatics procedures

ECF phylogenetic trees were built as described in Casas-Pastor et al.^17^. For Direct Coupling Analysis (DCA) protein sequences of members of groups ECF41 and ECF56 were retrieved from the most recent ECF classification^17^ and aligned using Clustal Omega 1.2.3^77^. DCA was performed using Gaussian DCA^78^ as described in Wu, et al.^23^. Calculation of differentially expressed genes: after trimming (ambiguous limit = 2 nt, quality limit = 0.01), RNA-seq generated reads were mapped onto the concatenated version of the reference genome (see *ChIP-sequencing* part in this section). Gene expression levels were determined based on the trancripts per Million (TPM). Expression with log2 fold change ≥ log2 (1.5) and q-value ≤ 0.05 or log2 fold change ≤ –log2 (1.5) and q-value ≤ 0.05 was considered as differentially expressed. Additional analyses of the generated sequence raw data were carried out using CLC Genomics Workbench 20 (Qiagen). Functional enrichment analyses: regulons were categorized according to their associated gene ontology IDs (GO) retrieved from the information available at Uniprot database. GSEA analysis was performed using the Broad Institute GSEA software^47^ using Gene set collections obtained by searching the *S.tsukubaensis* genome for homologues of proteins involved in sporulation and TCA through NCBI BLASTp analyses (https://blast.ncbi.nlm.nih.gov). The Δ*sigG1* vs WT full transcript list was ranked according to the log2 fold-change expression values and probed against the indicated gene signatures using the GSEA Pre-ranked mode with the following parameters: 10,000 permutations, classic scoring schem and meandiv normalization. ChIP-seq data analysis: reads resulting from paired-end sequencing were aligned to the genome of *S. tsukubaensis* using the bowtie2^79^ and further treated as described in Gallagher et al.^74^. Enrichment for the Δ*sigG1* control samples was subtracted from the enrichment in Δ*rsfG* samples. Significance of enrichment values were calculated assuming normal distribution of the enrichment values. Results were visualized in the Integrated Genome Browser^80^, or in the CLC Genomics Workbench 20 (Qiagen).  The reference genome was the concatenation of all contigs available on NCBI at the time (genome assembly ASM29715v2; WGS project AJSZ01). However, after this article was submitted for review, genome assembly ASM29715v2 was superceded by a new version (ASM29715v3) with an updated annotation (GenBank accession numbers: CP029157, CP029158 and CP029159). Whilst This does not affect the results or claims of this article, the locus tags that identify genes have changed. For instance, the locus tag for STSU_11560 has changed to STSU_011570 in the latest genome assembly version.

### Statistical analyses

For each experiment, we assayed at least three independent biological replicates. Statistical significance was addressed through the GraphPad Prism 8 software, according to the requirements of each data set.

## Supplementary information

Supplementary Information.

## Data Availability

Sequencing data was submitted in GEO under the following accession codes: GSE144815 for RNA-seq data and GSE144907 for ChIP-seq data.

## References

[1] Beites T (2015). *Streptomyces natalensis* programmed cell death and morphological differentiation are dependent on oxidative stress. Sci. Rep..

[2] Claessen D (2004). The formation of the rodlet layer of streptomycetes is the result of the interplay between rodlins and chaplins. Mol. Microbiol..

[3] Elliot MA (2003). The chaplins: a family of hydrophobic cell-surface proteins involved in aerial mycelium formation in *Streptomyces coelicolor*. Gene Dev..

[4] Flardh K, Buttner MJ (2009). *Streptomyces* morphogenetics: dissecting differentiation in a filamentous bacterium. Nat. Rev. Microbiol..

[5] McCormick JR, Flardh K (2012). Signals and regulators that govern *Streptomyces* development. FEMS Microbiol. Rev..

[6] Bush MJ, Tschowri N, Schlimpert S, Flärdh K, Buttner MJ (2015). c-di-GMP signalling and the regulation of developmental transitions in streptomycetes. Nat. Rev. Microbiol..

[7] Ulrich LE, Koonin EV, Zhulin IB (2005). One-component systems dominate signal transduction in prokaryotes. Trends Microbiol..

[8] Francis VI, Porter SL (2019). Multikinase networks: two-component signaling networks integrating multiple stimuli. Annu. Rev. Microbiol..

[9] Helmann JD (2019). Where to Begin? Sigma factors and the selectivity of transcription initiation in bacteria. Mol. Microbiol..

[10] Lonetto MA, Donohue TJ, Gross CA, Buttner MJ (2019). Discovery of the extracytoplasmic function σ factors. Mol. Microbiol..

[11] Helmann JD (2002). The extracytoplasmic function (ECF) sigma factors. Adv. Microb. Physiol..

[12] Lonetto M, Gribskov M, Gross CA (1992). The sigma 70 family: sequence conservation and evolutionary relationships. J. Bacteriol..

[13] Lane WJ, Darst SA (2006). The structural basis for promoter -35 element recognition by the group IV σ factors. PLoS Biol..

[14] Lin W (2019). Structural basis of ECF-σ-factor-dependent transcription initiation. Nat. Commun..

[15] Fang C (2019). Structures and mechanism of transcription initiation by bacterial ECF factors. Nucleic Acids Res..

[16] Gaballa A (2018). Modulation of extracytoplasmic function (ECF) sigma factor promoter selectivity by spacer region sequence. Nucleic Acids Res..

[17] Casas-Pastor, D. *et al.* Expansion and re-classification of the extracytoplasmic function (ECF) σ factor family. Preprint at 10.1101/2019.12.11.873521v2 (2019).

[18] Huang X, Pinto D, Fritz G, Mascher T (2015). Environmental sensing in Actinobacteria: a comprehensive survey on the signaling capacity of this phylum. J. Bacteriol..

[19] Staron A (2009). The third pillar of bacterial signal transduction: classification of the extracytoplasmic function (ECF) sigma factor protein family. Mol. Microbiol..

[20] Jogler C (2012). Identification of proteins likely to be involved in morphogenesis, cell division, and signal transduction in planctomycetes by comparative genomics. J. Bacteriol..

[21] Pinto D, Liu Q, Mascher T (2019). ECF σ factors with regulatory extensions: the one-component systems of the σ universe. Mol. Microbiol..

[22] Wecke T (2012). Extracytoplasmic function sigma factors of the widely distributed group ECF41 contain a fused regulatory domain. MicrobiologyOpen.

[23] Wu H (2019). The role of C-terminal extensions in controlling ECF σ factor activity in the widely conserved groups ECF41 and ECF42. Mol. Microbiol..

[24] Gómez-Santos N, Pérez J, Sánchez-Sutil MC, Moraleda-Muñoz A, Muñoz-Dorado J (2011). CorE from *Myxococcus xanthus* Is a copper-dependent RNA polymerase sigma factor. PLoS Genet..

[25] Marcos-Torres FJ, Pérez J, Gómez-Santos N, Moraleda-Muñoz A, Muñoz-Dorado J (2016). In depth analysis of the mechanism of action of metal-dependent sigma factors: characterization of CorE2 from *Myxococcus xanthus*. Nucleic Acids Res..

[26] Beinker P (2006). Crystal structures of SnoaL2 and AclR: two putative hydroxylases in the biosynthesis of aromatic polyketide antibiotics. J. Mol. Biol..

[27] Siitonen V, Blauenburg B, Kallio P, Mantsala P, Metsa-Ketela M (2012). Discovery of a two-component monooxygenase SnoaW/SnoaL2 involved in nogalamycin biosynthesis. Chem. Biol..

[28] Goutam K, Gupta AK, Gopal B (2017). The fused SnoaL-2 domain in the *Mycobacterium tuberculosis* sigma factor σjmodulates promoter recognition. Nucleic Acids Res..

[29] Liu Q, Pinto D, Mascher T (2018). Characterization of the widely distributed novel ECF42 group of extracytoplasmic function σ factors in *Streptomyces venezuelae*. J. Bacteriol..

[30] Krentz AJ (1994). Tacrolimus (FK506) versus cyclosporin in prevention of liver allograft rejection. Lancet.

[31] Jiang H, Yamamoto S, Nishikawa K, Kato R (1993). Anti-tumor-promoting action of FK506, a potent immunosuppressive agent. Carcinogenesis.

[32] Nagrani NK, Zito PM (2019). Topical tacrolimus (FK506, Protopic) in the treatment of atopic dermatitis. J. Dermatol. Nurses Assoc..

[33] Antelmann HAH, John D (2011). Thiol-based redox switches and gene regulation. Antioxid. Redox Sign..

[34] Weigt M, White RA, Szurmant H, Hoch JA, Hwa T (2009). Identification of direct residue contacts in protein–protein interaction by message passing. Proc. Natl. Acad. Sci..

[35] Bibb MJ, Domonkos Á, Chandra G, Buttner MJ (2012). Expression of the chaplin and rodlin hydrophobic sheath proteins in *Streptomyces venezuelae* is controlled by σ BldN and a cognate anti-sigma factor, RsbN. Mol. Microbiol..

[36] Asai K (2007). Regulatory role of RsgI in *sigI* expression in *Bacillus subtilis*. Microbiology.

[37] Bauer JS (2017). dRNA-seq transcriptional profiling of the FK506 biosynthetic gene cluster in *Streptomyces tsukubaensis* NRRL18488 and general analysis of the transcriptome. RNA Biol..

[38] Siranosian KJ, Ireton K, Grossman AD (1993). Alanine dehydrogenase (ald) is required for normal sporulation in *Bacillus subtilis*. J. Bacteriol..

[39] Bush MJ (2018). The actinobacterial WhiB-like (Wbl) family of transcription factors. Mol. Microbiol..

[40] Birch A, Häusler A, Hütter R (1990). Genome rearrangement and genetic instability in *Streptomyces* spp. J. Bacteriol..

[41] Cullum J, Altenbuchner J, Flett F, Piendl W (1986). DNA amplification and genetic instability in *Streptomyces*. Biotechnol. Genet. Eng. Rev..

[42] Leblond P, Decaris B (1994). New insights into the genetic of streptomyces instability. FEMS Microbiol. Lett..

[43] Volff J-N, Altenbuchner J (1998). Genetic instability of the *Streptomyces* chromosome. Mol. Microbiol..

[44] Zhang Z (2020). Antibiotic production in *Streptomyces* is organized by a division of labor through terminal genomic differentiation. Sci. Adv..

[45] Bailey TL (2009). MEME SUITE: tools for motif discovery and searching. Nucleic Acids Res..

[46] Salerno P (2013). Identification of new developmentally regulated genes involved in *Streptomyces coelicolor* sporulation. BMC Microbiol..

[47] Subramanian A (2005). Gene set enrichment analysis: a knowledge-based approach for interpreting genome-wide expression profiles. Proc. Natl. Acad. Sci. U. S. A..

[48] Neyfakh AA, Bidnenko VE, Chen LB (1991). Efflux-mediated multidrug resistance in *Bacillus subtilis*: similarities and dissimilarities with the mammalian system. Proc. Natl. Acad. Sci. U. S. A..

[49] Blin K (2017). antiSMASH 4.0—improvements in chemistry prediction and gene cluster boundary identification. Nucleic Acids Res..

[50] Andrews SC, Robinson AK, Rodriguez-Quinones F (2003). Bacterial iron homeostasis. FEMS Microbiol. Rev..

[51] Imlay J, Chin S, Linn S (1988). Toxic DNA damage by hydrogen peroxide through the Fenton reaction in vivo and in vitro. Science.

[52] Beites T (2011). Crosstalk between ROS homeostasis and secondary metabolism in *S. natalensis* ATCC 27448: modulation of pimaricin production by intracellular ROS. PLoS ONE.

[53] Manteca A, Yague P (2018). Streptomyces differentiation in liquid cultures as a trigger of secondary metabolism. Antibiotics (Basel).

[54] Martinez-Castro M (2013). Taxonomy and chemically semi-defined media for the analysis of the tacrolimus producer *Streptomyces tsukubaensis*. Appl. Microbiol. Biotechnol..

[55] Hahn JS, Oh SY, Roe JH (2002). Role of OxyR as a peroxide-sensing positive regulator in *Streptomyces coelicolor* A3(2). J. Bacteriol..

[56] Hu Y, Kendall S, Stoker NG, Coates ARM (2004). The *Mycobacterium tuberculosis sigJ* gene controls sensitivity of the bacterium to hydrogen peroxide. FEMS Microbiol. Lett..

[57] Yoo J-S, Oh G-S, Ryoo S, Roe J-H (2016). Induction of a stable sigma factor SigR by translation-inhibiting antibiotics confers resistance to antibiotics. Sci. Rep..

[58] González-Quiñónez N (2019). Cytosolic copper is a major modulator of germination, development and secondary metabolism in *Streptomyces coelicolor*. Sci. Rep..

[59] Chaplin AK, Tan BG, Vijgenboom E, Worrall JAR (2015). Copper trafficking in the CsoR regulon of *Streptomyces lividans*. Metallomics.

[60] Worrall JAR, Vijgenboom E (2010). Copper mining in *Streptomyces*: enzymes, natural products and development. Nat. Prod. Rep..

[61] Dwarakanath S (2012). Response to copper stress in *Streptomyces lividans* extends beyond genes under direct control of a copper-sensitive operon repressor protein (CsoR). J. Biol. Chem..

[62] Irving, H. & Williams, R. J. P. The stability of transition-metal complexes. *Journal of the Chemical Society (Resumed)* **0**, 3192–3210 (1953).

[63] Macomber L, Imlay JA (2009). The iron-sulfur clusters of dehydratases are primary intracellular targets of copper toxicity. Proc. Natl. Acad. Sci. U.S.A..

[64] Krumschnabel G, Manzl C, Berger C, Hofer B (2005). Oxidative stress, mitochondrial permeability transition, and cell death in Cu-exposed trout hepatocytes. Toxicol. Appl. Pharmacol..

[65] Fujimoto M (2012). Pleiotropic role of the Sco1/SenC family copper chaperone in the physiology of *Streptomyces*. Microb. Biotechnol..

[66] Traxler MF, Seyedsayamdost MR, Clardy J, Kolter R (2012). Interspecies modulation of bacterial development through iron competition and siderophore piracy. Mol. Microbiol..

[67] Gust B, Challis GL, Fowler K, Kieser T, Chater KF (2003). PCR-targeted *Streptomyces* gene replacement identifies a protein domain needed for biosynthesis of the sesquiterpene soil odor geosmin. Proc. Natl. Acad. Sci. U. S. A..

[68] Kieser T, Bibb M, Buttner M, Chater K, Hopwood DA (2000). Practical Streptomyces Genetics.

[69] Bush MJ, Chandra G, Findlay KC, Buttner MJ (2017). Multi-layered inhibition of *Streptomyces* development: BldO is a dedicated repressor of *whiB*. Mol. Microbiol..

[70] Pfaffl MW (2001). A new mathematical model for relative quantification in real-time RT-PCR. Nucleic Acids Res..

[71] Shin SH, Lim Y, Lee SE, Yang NW, Rhee JH (2001). CAS agar diffusion assay for the measurement of siderophores in biological fluids. J. Microbiol. Methods.

[72] Yang JH (2006). *Bradyrhizobium japonicum* senses iron through the status of haem to regulate iron homeostasis and metabolism. Mol. Microbiol..

[73] Ordonez-Robles M, Santos-Beneit F, Rodriguez-Garcia A, Martin JF (2017). Analysis of the Pho regulon in *Streptomyces tsukubaensis*. Microbiol. Res..

[74] Gallagher KA (2019). c-di-GMP arms an anti-σ to control progression of multicellular differentiation in *Streptomyces*. Mol. Cell.

[75] Karimova G, Pidoux J, Ullmann A, Ladant D (1998). A bacterial two-hybrid system based on a reconstituted signal transduction pathway. Proc. Natl. Acad. Sci. U. S. A..

[76] Miller, J. H. *Experiments in Molecular Genetics*. (Cold Spring Harbor Laboratory Press, 1972).

[77] Sievers F (2011). Fast, scalable generation of high-quality protein multiple sequence alignments using Clustal Omega. Mol. Syst. Biol..

[78] Baldassi C (2014). Fast and accurate multivariate gaussian modeling of protein families: predicting residue contacts and protein-interaction partners. PLoS ONE.

[79] Langmead B, Salzberg SL (2012). Fast gapped-read alignment with Bowtie 2. Nat. Methods.

[80] Nicol JW, Helt GA, Blanchard SG, Raja A, Loraine AE (2009). The Integrated Genome Browser: free software for distribution and exploration of genome-scale datasets. Bioinformatics.

[81] Crooks GE, Hon G, Chandonia J-M, Brenner SE (2004). WebLogo: a sequence logo generator. Genome Res..

[82] Barreiro C (2012). Draft genome of *Streptomyces tsukubaensis* NRRL 18488, the producer of the clinically important immunosuppressant tacrolimus (FK506). J. Bacteriol..

[83] Datsenko KA, Wanner BL (2000). One-step inactivation of chromosomal genes in *Escherichia coli* K-12 using PCR products. Proc. Natl. Acad. Sci. U. S. A..

[84] Paget MSB, Chamberlin L, Atrih A, Foster SJ, Buttner MJ (1999). Evidence that the extracytoplasmic function sigma factor σE is required for normal cell wall structure in *Streptomyces coelicolor* A3(2). J. Bacteriol..

[85] Sherwood EJ, Hesketh AR, Bibb MJ (2013). Cloning and analysis of the planosporicin lantibiotic biosynthetic gene cluster of *Planomonospora alba*. J. Bacteriol..

