# Supplementary material for: The novel ECF56 SigG1-RsfG system modulates morphological differentiation and metal-ion homeostasis in Streptomyces tsukubaensis
Source: Sci Rep. 2020 Dec 10;10:21728. doi: 10.1038/s41598-020-78520-x (PMC7730460; doi:10.1038/s41598-020-78520-x)
Supplement: Supplementary file 1 — Supplementary Information. [file 41598_2020_78520_MOESM1_ESM.pdf]

Supplementary Information

The novel ECF56 SigG1-RsfG system modulates morphological differentiation and metal-ion homeostasis in *Streptomyces tsukubaensis*

Rute Oliveira [1,2,3], Matthew J. Bush [4], Sílvia Pires [2†], Govind Chandra [4], Delia Casas-Pastor [5], Georg Fritz [5††], and Marta V. Mendes [1,2*],

[1]Bioengineering and Synthetic Microbiology Group, i3S- Instituto de Investigação e Inovação em Saúde, Universidade do Porto, Porto, Portugal

[2]IBMC, Instituto de Biologia Molecular e Celular, Universidade do Porto, Porto, Portugal

[3]Programa Doutoral em Biologia Molecular e Celular (MCBiology), ICBAS, Instituto de Ciências Biomédicas Abel Salazar, Universidade do Porto, Porto, Portugal

[4]Department of Molecular Microbiology, John Innes Centre, Norwich Research Park, Norwich NR4 7UH, United Kingdom

[5]LOEWE -Zentrum für Synthetische Mikrobiologie, Philipps -Universität Marburg, 35032 Marburg, Germany

[*] Corresponding author: mvm@ibmc.up.pt

[†] Current address: Weill Cornell University, New York, NY 10021, United States of America

[††] Current address: School for Molecular Sciences, University of Western Australia, Perth 6009, Australia

Supplementary Table S1. Oligonucleotides used in this study

Primer	Sequence 5' - 3'	Use
Red sigG1 F	GTGTTTCCCGGTGGTGCTGCTGATACGAAGAGAGGTATGATTCCGGGGAT<br>CCGTCGACC	Redirect^{TM} gene KO
Red_sigG1_R	CACGGGAAAGGAATCGGCGGACTGTTGTGACCCGGGTCATGTAGGCTGGA<br>GCTGGTTC	Redirect^{TM} gene KO
Red_rsfG_F	GCGCACCCTCACCACCACGGCAAAGGACGCAGAACCATGATTCCGGGGAT<br>CCGTCGACC	Redirect^{TM} gene KO
Red_rsfG_R	ACGGGACGTCCGGGCCGCCGTCGCTCCGGCCGGTGTTCATGTAGGCTGGA<br>GCTGCTTC	Redirect^{TM} gene KO
Red_conf_sigG1_F	CGGAAGTGCAGATGCTCGGC	Redirect^{TM} gene KO
Red_conf_sigG1_R	AACCGAGCAAAGGCATGACC	Redirect^{TM} gene KO
Red_conf_rsfG_F	TGCGGCTCCCACGGGACGT	Redirect^{TM} gene KO
Red_conf_rsfG_R	GCACCCTCACCACCACGGCA	Redirect^{TM} gene KO
RT_sigG1_F	GACGGTCATATCGAGGGCGT	RT-qPCR analyses
RT_sigG1_R	GTGATCCAGCATCTGCCGCC	RT-qPCR analyses
RT_rsfG_F	CCGAACTGCTCCAGGGCGAT	RT-qPCR analyses
RT_rsfG_R	GGACAGGCTGATCGGCACCT	RT-qPCR analyses
RT_oxyR_F	CAGCCCAGCCTGTCGCAACT	RT-qPCR analyses
RT_oxyR_F	AGAGCGCGGGCTGACTCATG	RT-qPCR analyses
RT_hrdB_F	GCGGCACTGACCATCAGCGT	RT-qPCR reference gene
RT_hrdB_F	GATTCCGCCAACCCAGTGGA	RT-qPCR reference gene
RT_rpsP_F	GCGCCGACGGAAAGCCAGTA	RT-qPCR reference gene
RT_rpsP_R	CCATCGAGGAGATCGGCCTG	RT-qPCR reference gene
GSP1_sigG1	CAGGGCGAGTGCGGACAGGT	TSS mapping
GSP3_sigG1	GATGCTCGGCTCCTTCACC	TSS mapping
GSP1_rsfG	CAGATGGTGAGGGTGTCGCC	TSS mapping
GSP2_rsfG	CGTAGTAGCGGGAGCGGATGT	TSS mapping
5'RACE AAP	GGCCACGCGTCGACTAGTACGGGIIGGGIIGGGIIG	TSS mapping
5´RACE AUAP	GGCCACGCGTCGACTAGTAC	TSS mapping
KpnI_sigG1_F	CGGGTACCTATGAGCGACCAGGGTGG	Bacterial two-hybrid
EcoRI_sigG1_stop_R	AGGAATTCGGCGGACTGTTGTGACC	Bacterial two-hybrid
EcoRI_sigG1_R	CTGAATTCTCCGCCAGGGTGGGG	Bacterial two-hybrid
KpnI_rsfG_F	CTGGTACCCATGAACGACACCACCGC	Bacterial two-hybrid
EcoRI_rsfG_stop_R	ACGAATTCACGGGGTGATCGGGAC	Bacterial two-hybrid
EcoRI_rsfG_R	ATGAATTCACCGCGACCCGGGTCA	Bacterial two-hybrid
KpnI_SigG1SnoaL2	GTGGTACCTGCGGCCACCGAGCCCAGCG	Bacterial two-hybrid
EcoRI_SigG1r2r4	TAGAATTCCGCACCCCACTCCGA	Bacterial two-hybrid

EcoRI_rsfGNT	ATGAATTCGGTGCCGATCAGCCTGTC	Bacterial two-hybrid
T18seq_F	GTGTGGAATTGTGAGCGGAT	Bacterial two-hybrid
T18seq_R	TTCCACAACAAGTCGATGCG	Bacterial two-hybrid
T25seq_F	CGGTGACCAGCGGCGATT	Bacterial two-hybrid
T25seq_R	GGCGATTAAGTTGGGTAACGCC	Bacterial two-hybrid
NT25seq_F	CCCCAGGCTTTACACTTTATGC	Bacterial two-hybrid
NT25seq_R	TTGATGCCATCGAGTACGGCT	Bacterial two-hybrid
T18Cseq_F	GTGCCGAGCGGACGTTCGA	Bacterial two-hybrid
T18Cseq_R	CTTAACTATGCGGCATCAGAGC	Bacterial two-hybrid
rsfG_MCS1_F	ACACAGAATTCATGAACGACACCACCGCA	Protein co-expression
rsfG_MCS1_R	CATAAGCTTTCACCGGTAGTAGGCCGGGGA	Protein co-expression
sigG1_MCS2_F	GAACCCATATGAGCGACCAGGGTGGC	Protein co-expression
sigG1_MCS2_R	CAGGTACCATCGGCGGACTGTTGTGA	Protein co-expression
SigG1_SpeI_F	GCACTAGTTCACGGGAAAGGAATCGG	*psigG1-sigG1* expression
SigG1_SpeI_R	GCACTAGTAGAGCAGCACCTTCCGGG	*psigG1-sigG1* expression

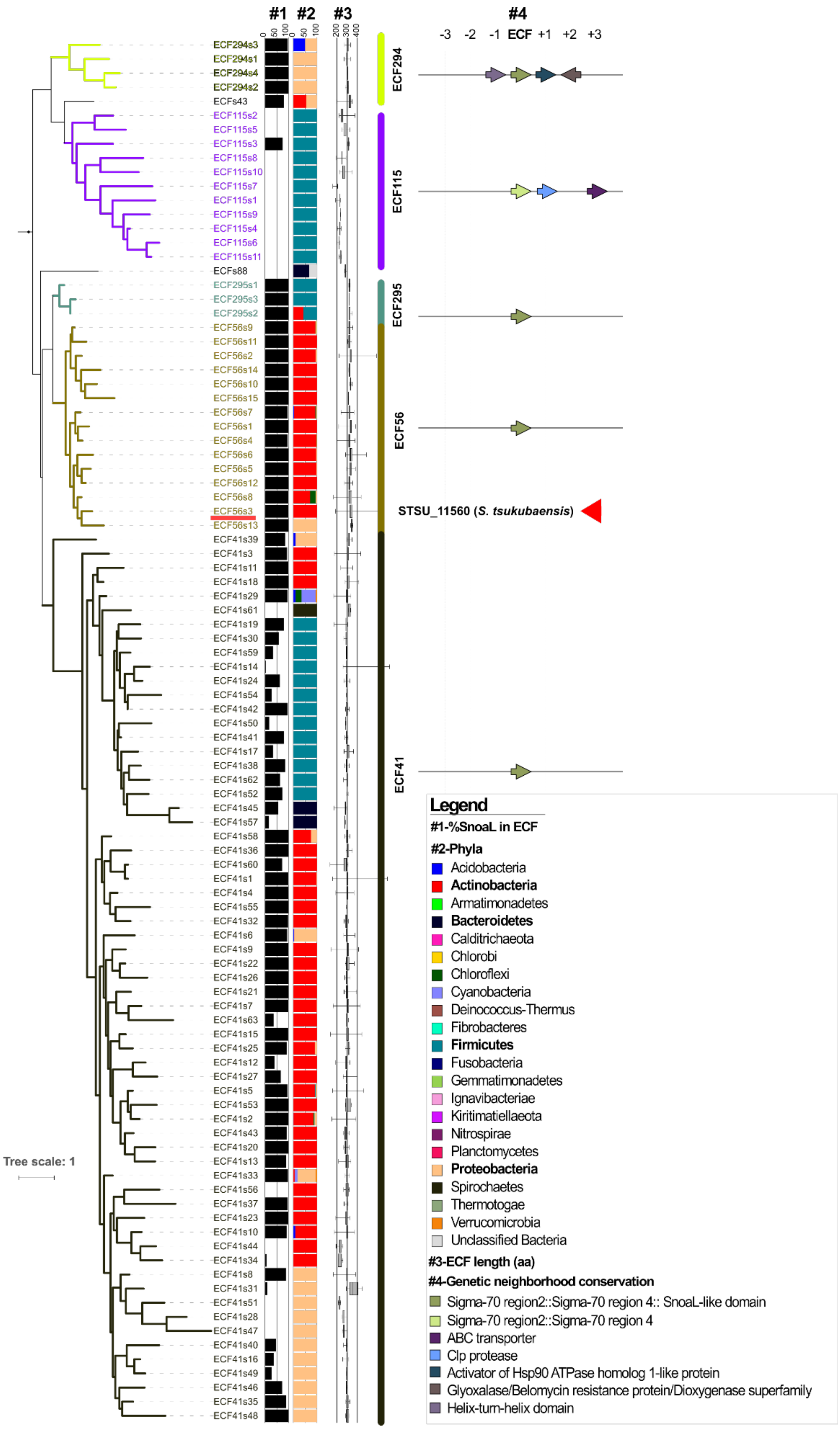
#1
#2
#3
#4
0 50 100
0 50 100
200 300 400
-3 -2 -1 ECF +1 +2 +3
ECF294s3
ECF294s1
ECF294s4
ECF294s2
ECFs43
ECF115s2
ECF115s5
ECF115s3
ECF115s8
ECF115s10
ECF115s7
ECF115s1
ECF115s9
ECF115s4
ECF115s6
ECF115s11
ECFs88
ECF295s1
ECF295s3
ECF295s2
ECF56s9
ECF56s11
ECF56s2
ECF56s14
ECF56s10
ECF56s15
ECF56s7
ECF56s1
ECF56s4
ECF56s6
ECF56s5
ECF56s12
ECF56s8
ECF56s3
ECF56s13
ECF41s39
ECF41s3
ECF41s11
ECF41s18
ECF41s29
ECF41s61
ECF41s19
ECF41s30
ECF41s59
ECF41s14
ECF41s24
ECF41s54
ECF41s42
ECF41s50
ECF41s41
ECF41s17
ECF41s38
ECF41s62
ECF41s52
ECF41s45
ECF41s57
ECF41s58
ECF41s36
ECF41s60
ECF41s1
ECF41s4
ECF41s55
ECF41s32
ECF41s6
ECF41s9
ECF41s22
ECF41s26
ECF41s21
ECF41s7
ECF41s63
ECF41s15
ECF41s25
ECF41s12
ECF41s27
ECF41s5
ECF41s53
ECF41s2
ECF41s43
ECF41s20
ECF41s13
ECF41s33
ECF41s56
ECF41s37
ECF41s23
ECF41s10
ECF41s44
ECF41s34
ECF41s8
ECF41s31
ECF41s51
ECF41s28
ECF41s47
ECF41s40
ECF41s16
ECF41s49
ECF41s46
ECF41s35
ECF41s48
ECF294
ECF115
ECF295
ECF56
ECF41
STSU_11560 (S. tsukubaensis)
Tree scale: 1
Legend
#1-%SnoaL in ECF
#2-Phyla
Acidobacteria
Actinobacteria
Armatimonadetes
Bacteroidetes
Calditrichaeota
Chlorobi
Chloroflexi
Cyanobacteria
Deinococcus-Thermus
Fibrobacteres
Firmicutes
Fusobacteria
Gemmatimonadetes
Ignavibacteriae
Kiritimatiellaeota
Nitrospirae
Planctomycetes
Proteobacteria
Spirochaetes
Thermotogae
Verrucomicrobia
Unclassified Bacteria
#3-ECF length (aa)
#4-Genetic neighborhood conservation
Sigma-70 region2::Sigma-70 region 4:: SnoaL-like domain
Sigma-70 region2::Sigma-70 region 4
ABC transporter
Clp protease
Activator of Hsp90 ATPase homolog 1-like protein
Glyoxalase/Belomycin resistance protein/Dioxygenase superfamily
Helix-turn-helix domain

Fig. S1 - **SigG1 phylogenetic distribution within the bacteria ECF group classification.** The most recent classification distributes ECFs throughout a total of 157 families (see main text for references). Each family is labeled with a different colour. Phylogenetic analyses indicate that SnoaL2 containing ECFs (#1) are present in various phyla (#2 and indicated on the right) and are distributed throughout 5 ECF families. SigG1 is included in the ECF56 family (highlighted in dark gold), which includes proteins with the conserved σ2 and σ4 domains followed by a CT SnoaL2 domain, exclusively. ECF sigma factors were collected from 14,483 bacterial genomes. The conserved position of the ECF relatively to other known proteins is depicted as *Genomic Neighbourhood Conservation* (#4).

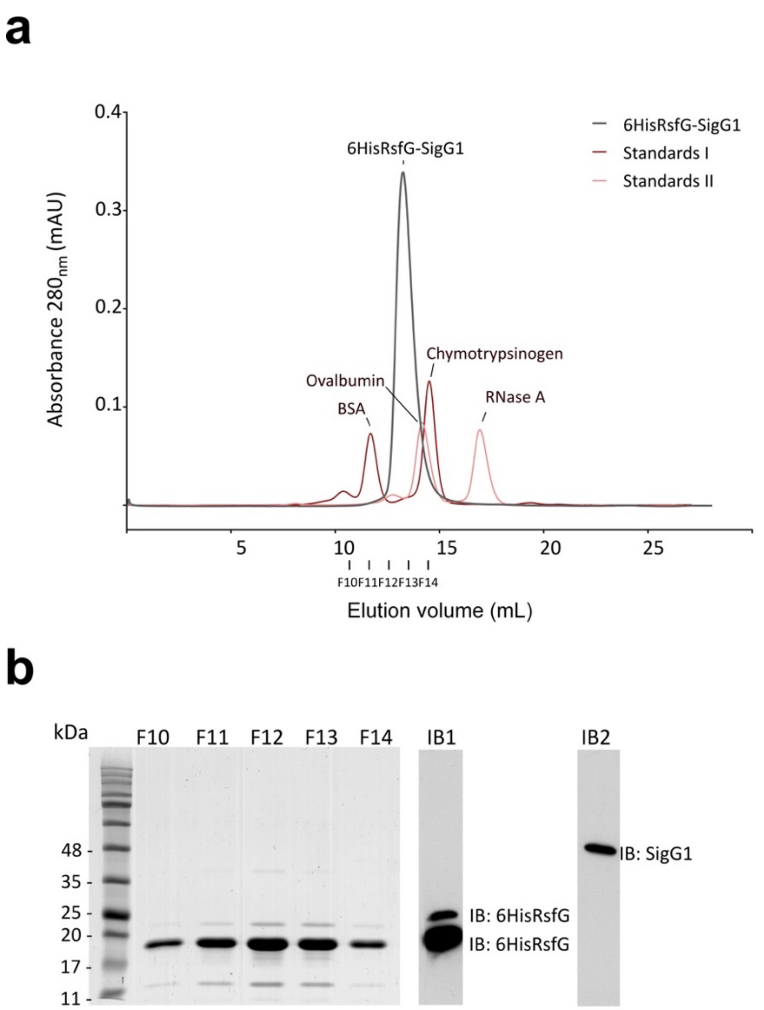

Fig S2 - Size exclusion chromatography of the eluted 6His-RsfG-SigG1 complex purified by Ni-NTA affinity chromatography. (A) Chromatogram of the 6HisRsfG-SigG1 complex eluted from the Superose 12 SEC column. Protein standards of known molecular weights were used to generate a calibration curve and for probe the molecular weight of the eluted complex. Elution profile was monitored at 280 nm and 1 mL fractions were collected. BSA: 66 kDA; Ovalbumin: 44 kDA; Chymotrypsinogen: 25 kDa; Ribonuclease A: 13.7 kDa (B) Eluted fractions were analysed by SDS-PAGE stained with Coomassie blue. 15 µL of each fraction was loaded into the gel. The presence of the two proteins (6His-RsfG and SigG1) was confirmed by immunoblot using and antibody against the 6His-tag (IB1) and a polyclonal antibody anti-SigG1 (IB2). Original uncropped images are presented in Supplementary Fig. S11.

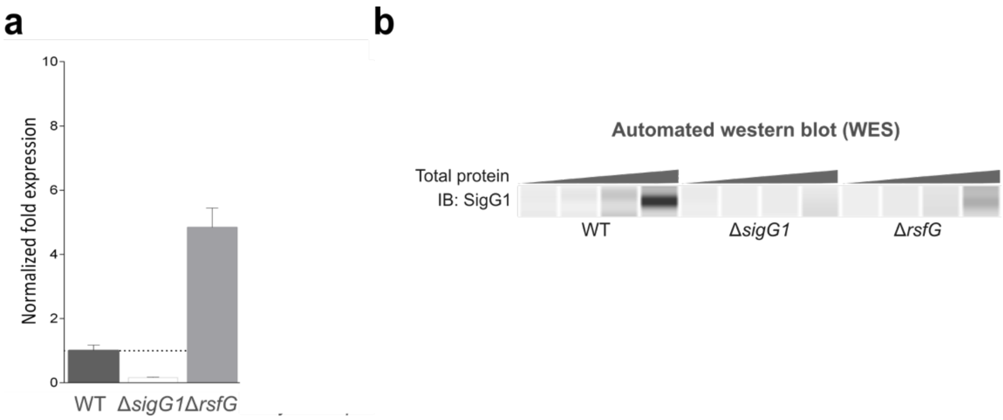

Fig. S3 - *sigG1* gene expression in *S. tsukubaensis*. (**A**) Transcript levels in *sigG1*-related mutant strains evaluated by RT-qPCR. (**B**) SigG1 protein expression in the wild-type and the *rsfG null* mutant was detected by automated western blot (WES Simple) using a polyclonal anti-SigG1 antibody. Original uncropped images are presented in Supplementary Fig. S11c.

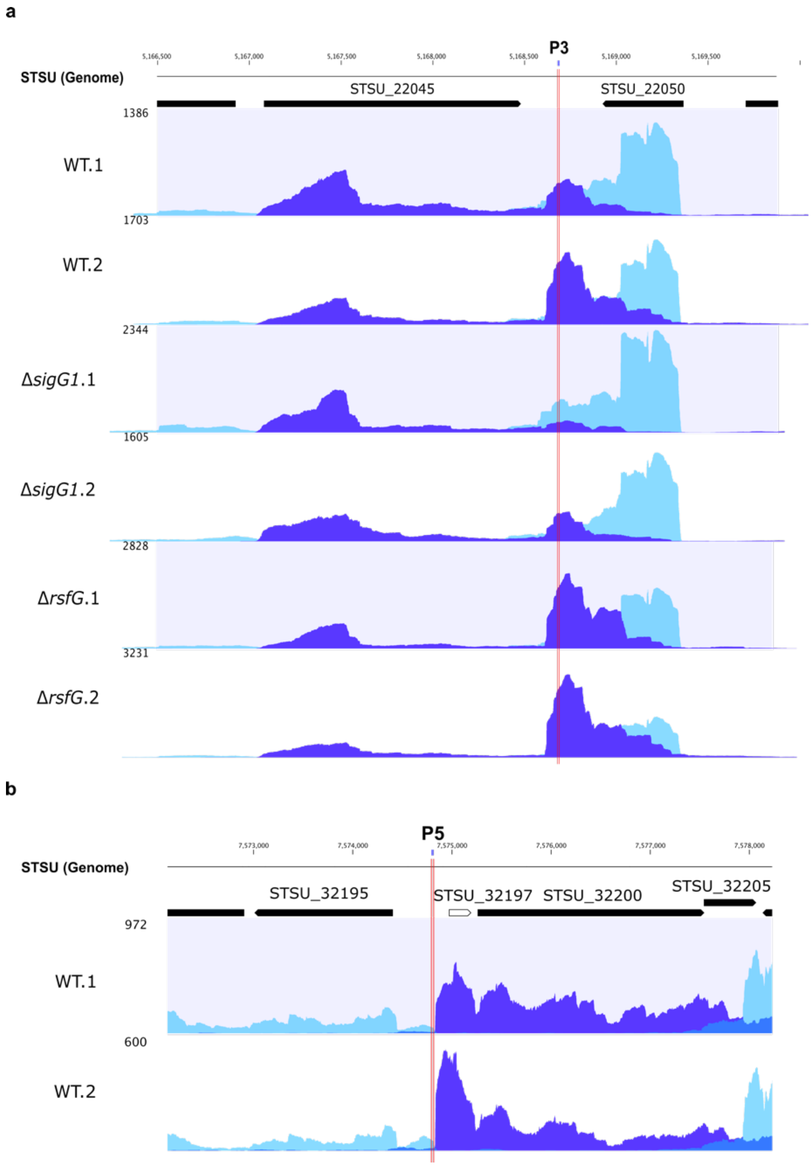

Fig. S4 - Visualisation of RNA-seq coverage in selected genomic regions. (**A**) Reads coverage of the genomic region that includes the ChIP-seq peak 3 (P3) showing a putative antisense RNA for *STSU_22050*, regulated by SigG1. *STSU_22050* codes for a hypothetical protein. (**B**) Reads coverage of the ChIP-seq peak 5 (P5) region showing the presence of a putative a new gene (*STSU_32197*), encoding a glycine-rich transmembrane hypothetical protein, upstream of Cu^{2+}-exporting ATPase (STSU_32200). The y-axis shows the read count. For each strain, two biological replicas are shown. Dark blue indicates coverage of the forward reads and light blue reads mapping in the reverse orientation. NGS data was obtained by RNA-seq and SigG1 native ChIP-seq experiments.

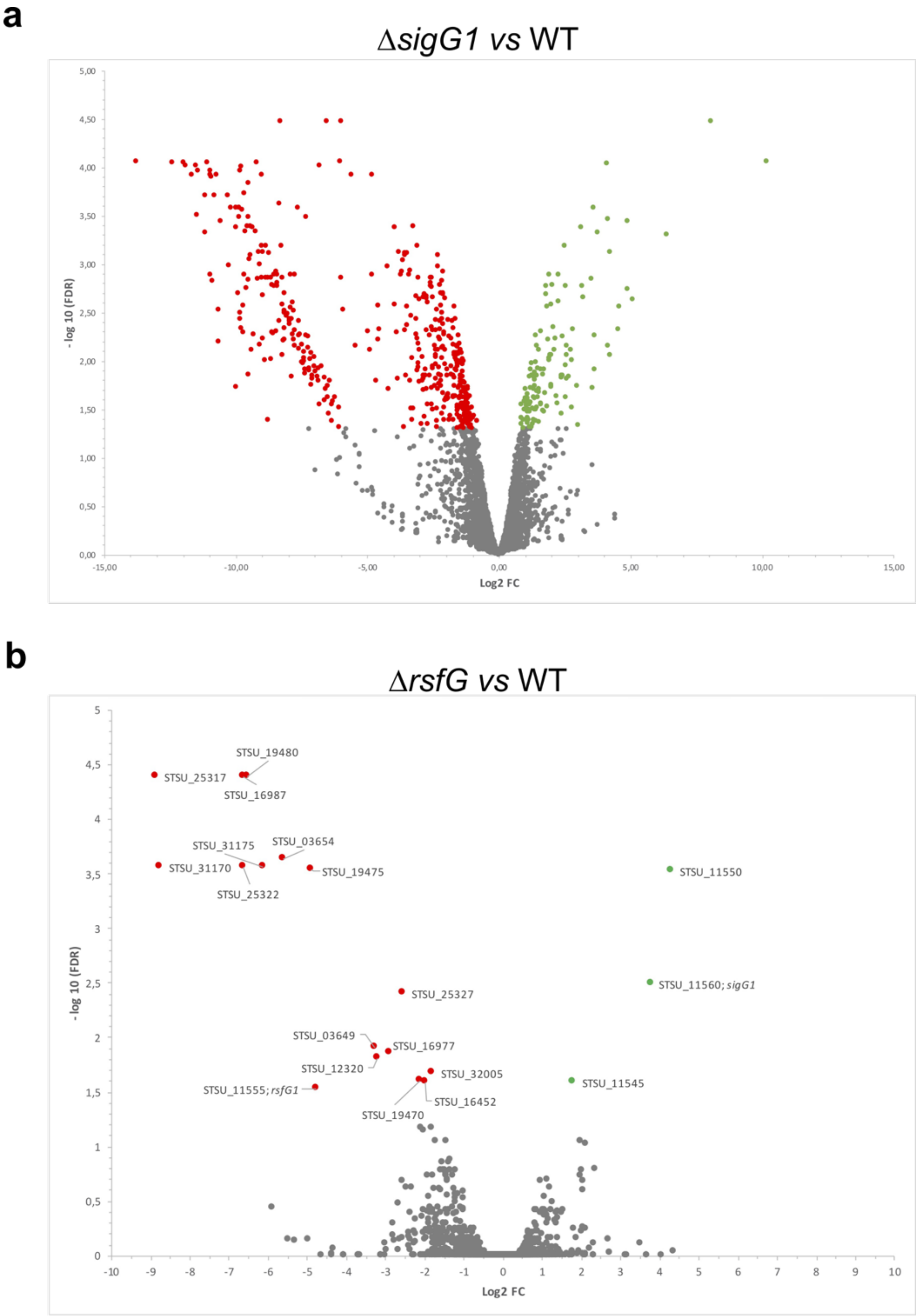

Fig. S5 - **Volcano plot representation.** Differential expression genes (DEGs) in the Δ*sigG1 vs* WT comparison (A) and Δ*rsfG vs* WT (B). X-axis shows $\log_2$ FC and y-axis displays the $-\log_{10}$ (FDR). Green dots represent up-regulated DEGs (FDR < 0.05), red dots down-regulated DEGs (FDR < 0,05) and grey dots not-DEGs (FDR > 0.05).

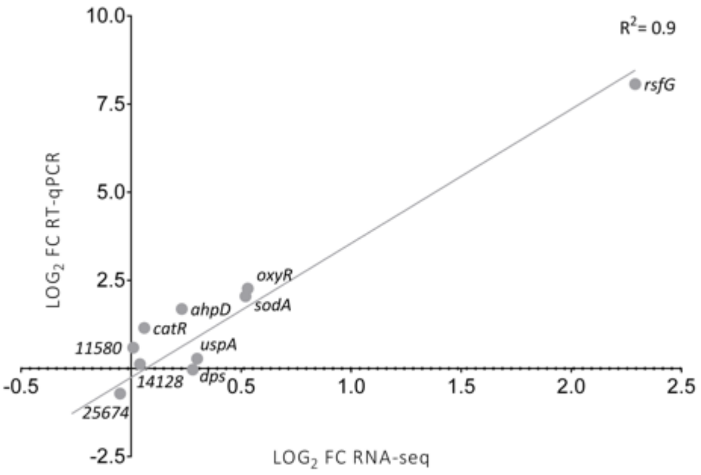

Fig. S6 - Correlation between RNA-seq fold expression and RT-qPCR. Data represented as Log2 of fold change between in vitro gene expression of *S. tsukubaensis* WT and Δ*sigG1* as measured by RNA-seq. Results are representative of three independent experiments; r^2=0,9

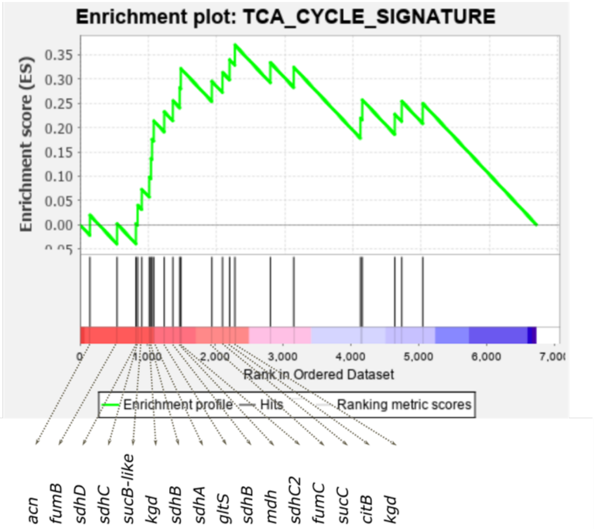

Fig. S7 - Gene set enrichment analysis (GSEA) in the Δ*rsfG* mutant. Molecular signatures for specific cellular pathways were scanned against the whole transcriptome of the wild-type and the Δ*rsfG* obtained by RNA-sequencing. GSEA Prerank analysis of the molecular signature of proteins involved in the TCA cycle in *Streptomyces*. Enrichment of candidate gene signatures in Δ*rsfG* as compared to the wild-type are depicted. Normalized Enrichment Score (NES)= 2.17; FDR q-value= 0.002.

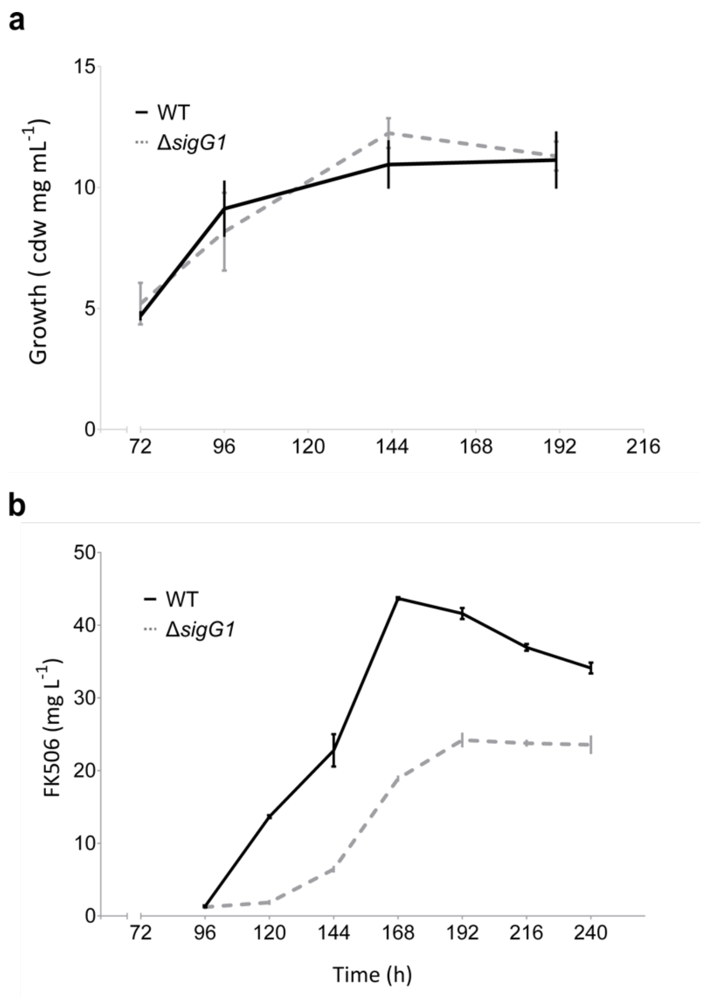

Fig. S8 - Growth (A) and FK506 biosynthesis (B) of wild-type (black line) and Δ*sigG1* strains (dashed grey line). Growth was determined by cell dry weight and FK506 production was quantified by high performance liquid chromatography (HPLC). Values are representative of at least three independent experiments.

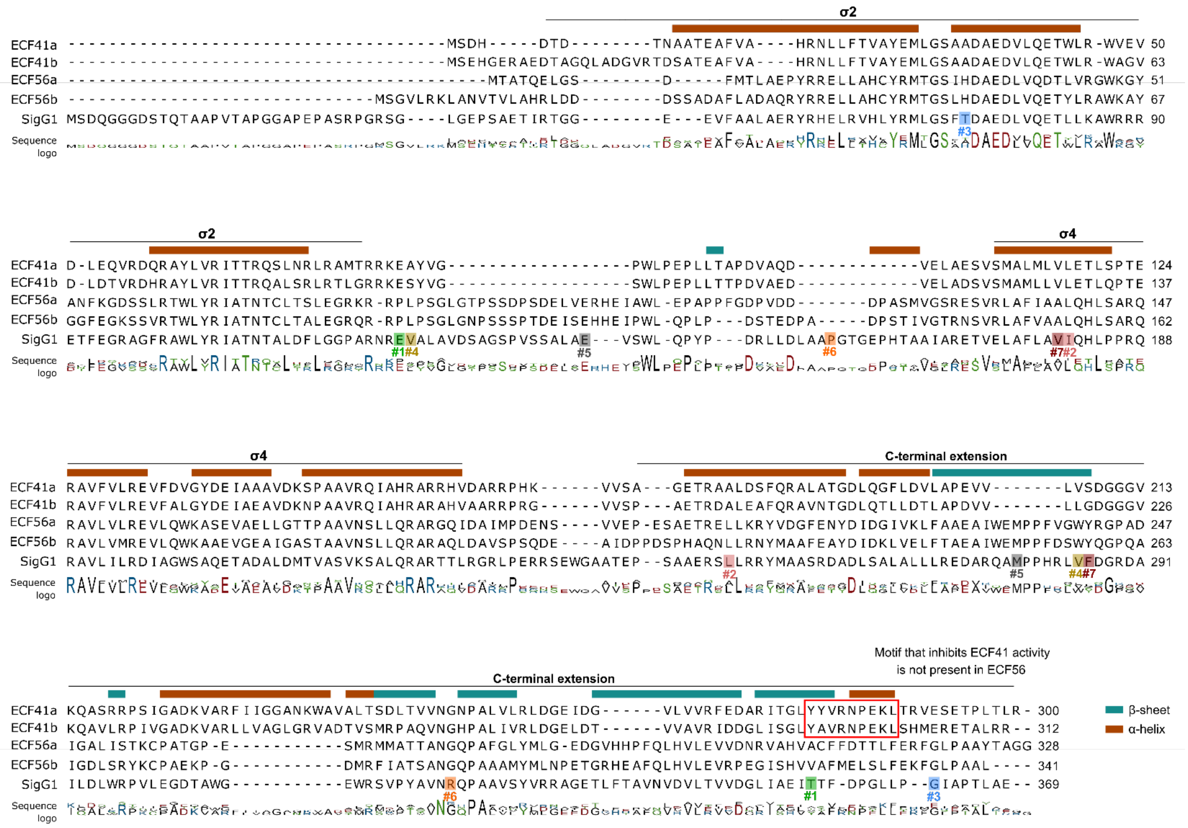

Fig. S9 - Multiple sequence alignment of ECF41 activity inhibiting motif. Selected sequences for sigma factors representatives of the ECF41 and ECF56 families were aligned as described in [1]. The figure depicts the consensus NPDKL motif of the NTF2-like CT region required to interact with the σ_2 and σ_4 linker and inhibit the ECF activity[2]. The conserved motif in ECF41 is not present in ECF56. ECF domains and secondary structure are shown. DCA predictions are mapped for selected residues. Pairs of contacting residues are highlighted in colour.

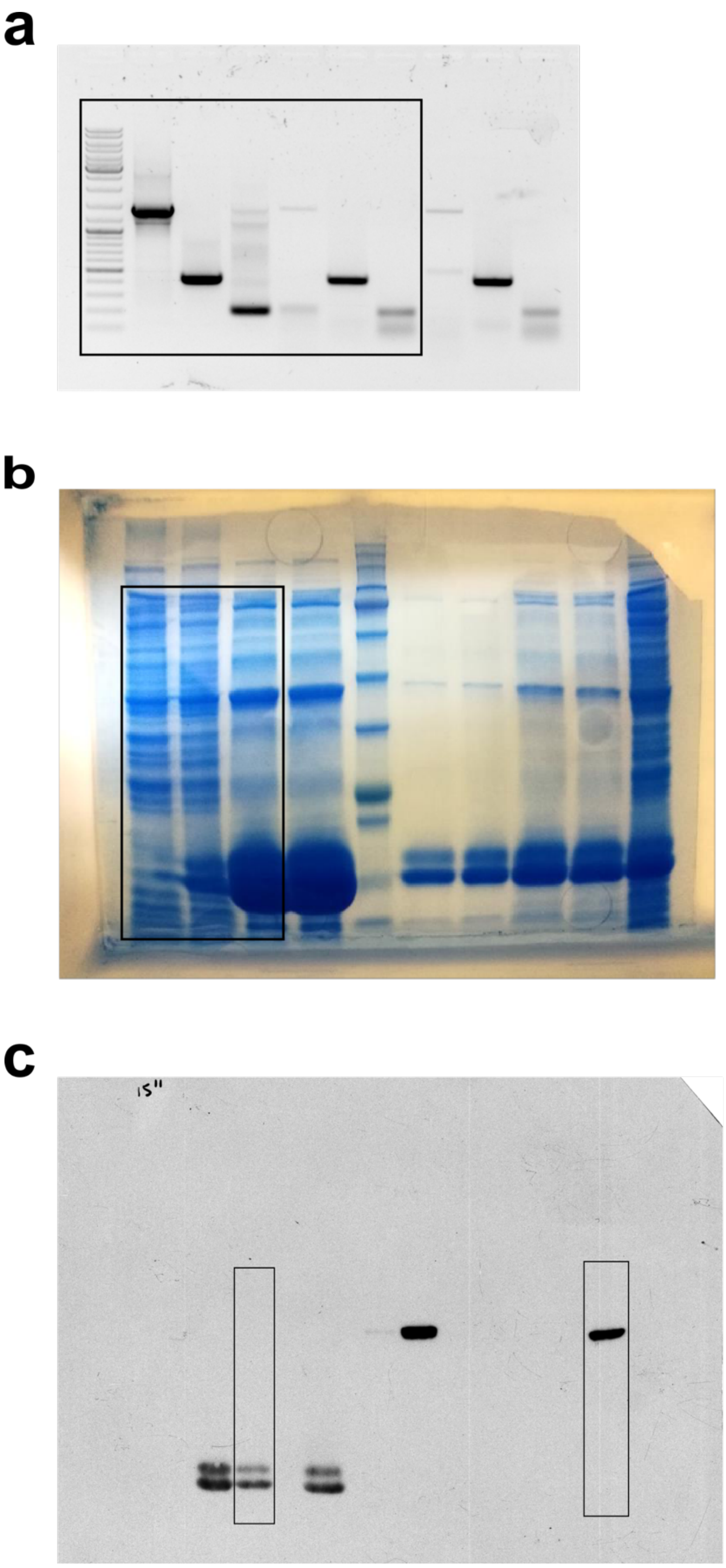

Fig. S10 – Unprocessed images of PCR gel (A) SDS-PAGE (B) and immunoblot (C) displayed in Fig. 2. Cropped regions are indicated by a box in each image.

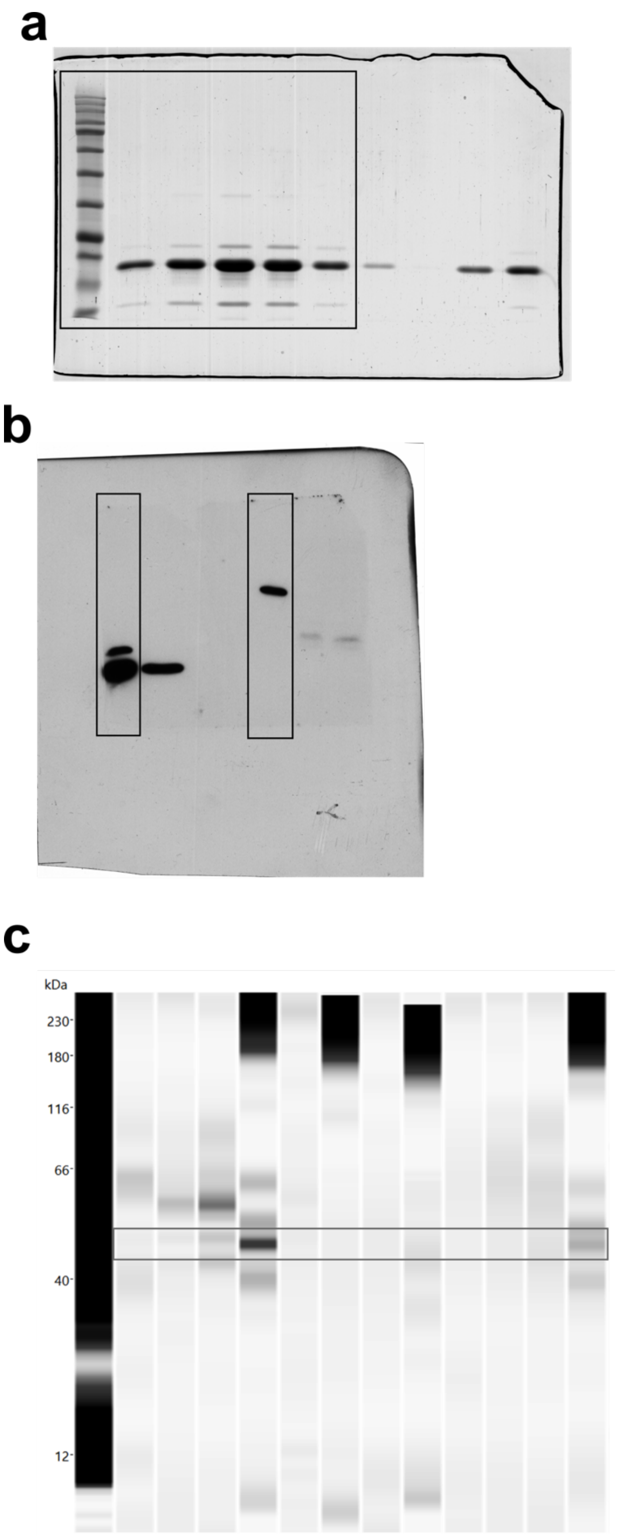

Fig. S11 – Unprocessed images of (A) SDS-PAGE, (B) immunoblot displayed in Fig. S2B and (C) automated western blot (WES Simple) displayed in Fig. S3B. Cropped regions are indicated by a box in each image.

References:

1 Casas-Pastor, D. *et al.* Expansion and re-classification of the extracytoplasmic function (ECF) σ factor family. Preprint at https://www.biorxiv.org/content/10.1101/2019.1112.1111.873521v873522 (2019).

2 Wecke, T. *et al.* Extracytoplasmic function sigma factors of the widely distributed group ECF41 contain a fused regulatory domain. *MicrobiologyOpen* **1**, 194-213 (2012).
